# *λ*-Deformation: A Canonical Framework for Statistical Manifolds of Constant Curvature

**DOI:** 10.3390/e24020193

**Published:** 2022-01-27

**Authors:** Jun Zhang, Ting-Kam Leonard Wong

**Affiliations:** 1 Department of Psychology, University of Michigan, Ann Arbor, MI 48109-1109, USA; 2 Department of Statistics, University of Michigan, Ann Arbor, MI 48109-1109, USA; 3 Department of Statistical Sciences, University of Toronto, Toronto, ON M5S 1A1, Canada; tkl.wong@utoronto.ca

**Keywords:** Legendre duality, λ-duality, λ-exponential family, λ-mixture family, conformal Hessian, constant curvature space

## Abstract

This paper systematically presents the λ-deformation as the canonical framework of deformation to the dually flat (Hessian) geometry, which has been well established in information geometry. We show that, based on deforming the Legendre duality, all objects in the Hessian case have their correspondence in the λ-deformed case: λ-convexity, λ-conjugation, λ-biorthogonality, λ-logarithmic divergence, λ-exponential and λ-mixture families, etc. In particular, λ-deformation unifies Tsallis and Rényi deformations by relating them to two manifestations of an identical λ-exponential family, under subtractive or divisive probability normalization, respectively. Unlike the different Hessian geometries of the exponential and mixture families, the λ-exponential family, in turn, coincides with the λ-mixture family after a change of random variables. The resulting statistical manifolds, while still carrying a dualistic structure, replace the Hessian metric and a pair of dually flat conjugate affine connections with a conformal Hessian metric and a pair of projectively flat connections carrying constant (nonzero) curvature. Thus, λ-deformation is a canonical framework in generalizing the well-known dually flat Hessian structure of information geometry.

## 1. Introduction

Information geometry is a differential-geometric framework for studying finite-dimensional statistical models that coherently integrates the following notions:

(i) A differentiable manifold M consisting of probability density functions or finite measures on a common sample space;
(ii) A divergence function $D[p||p^{\prime }]$ that defines an asymmetric proximity between points *p*, $p^{\prime }$ in M;
(iii) A Riemannian metric g plus a pair of torsion-free dual (conjugate) affine connections $\nabla ,\nabla^{*}$ on M.

For completeness, we recall that a pair of affine connections ∇, $\nabla^{*}$ on M are said to be dual (or conjugate) with respect to a Riemannian metric g if for any vector fields *X*, *Y*, and *Z* on M, one has:

(1) $$Zg\left(X,Y\right)=g\left(\nabla_{Z}X,Y\right)+g\left(X,\nabla_{Z}^{*}Y\right).$$

Here, $\left(M,g,\nabla ,\nabla^{*}\right)$ is called a *dualistic structure*. When *D* is the Kullback–Leibler divergence (or more generally, *f*-divergence), the induced Riemannian metric g is the Fisher–Rao metric, and the induced cubic form $C=\nabla^{*}-\nabla$ is the Amari–Chentsov tensor [1]. It can be shown that the Fisher–Rao metric and the Amari–Chentsov tensor are unique invariants, of respectively second and third orders, under sufficient statistics on the manifold M [2].

Geometrically, the standard model (denoted the S-model in this paper) uses a pair of affine connections that are torsion-free, though in general, they are not curvature-free. An alternative, “partially flat” model (denoted the P-model in this paper) was recently investigated in [3], leading to the notion of “statistical mirror symmetry” [4]. Under the P-model, the affine connections ∇ and $\nabla^{*}$ are allowed to carry torsion, but are both curvature-free. See [4] for the geometric properties of the P-model leading to a symplectic-to-complex correspondence characteristic of mirror Calabi–Yau manifolds studied in string theory and mathematical physics.

Within the usual S-model, a special case is the *dually flat geometry* where the Riemannian metric can be expressed under special coordinate systems as a Hessian metric. Two prominent examples are the exponential family and the mixture family, where the Hessian metric coincides with the Fisher–Rao metric. The Hessian geometry is said to be dually flat because the Riemann curvature tensors of both the primal and the dual connections vanish; the corresponding primal and dual affine coordinate systems are linked via Legendre transformations by a pair of convex potentials. For an exponential family, these coordinates are precisely the natural (canonical) and mixture (expectation) coordinate systems, respectively. Note that the Hessian metric itself is not flat as its Levi-Civita connection contains curvature in general.

Between the well-understood dually flat Hessian geometry and the full-blown S-model, there is a wide range of geometries capturing various probability models. Of special interest are generalizations of the exponential family, namely *deformed exponential families*. The ϕ-exponential family was introduced in the context of statistical physics [5]; it was later shown [6] to be equivalent to the *U*-model [7] motivated by applications in machine learning—[6] revealed that both the ϕ- and *U*-models can be generated from the (ρ,τ)-model [8] through the mechanism of “gauge selection”. The (ρ,τ)-metric generalizes the Fisher–Rao metric and may lead to a conformal Hessian metric for a ϕ-exponential family. However, the connections are typically not curvature-free unless a special type of gauge is selected; this underlies the geometric characterization of the *q*-exponential model of Tsallis by [9,10,11].

In recent years, the second author [12], motivated by previous works with Pal on mathematical finance and optimal transport [13,14,15,16], studied a class of deformed exponential families generating *constant curvatures* through the use of a new divergence function called *logarithmic divergence*. By constant (information geometric) curvature, we mean that both the primal and dual Riemann curvature tensors have (the same) constant sectional curvature with respect to g. In [17], the present authors developed a unified framework, based on the notions of *λ-duality* and the *λ-exponential family*, which appears to provide a *canonical* extension of the dually flat geometry to the constant curvature case. Previously, statistical manifolds with constant curvature were studied using the abstract tools of affine differential geometry; see, e.g., [1,18] (also see [19]). Our framework provides a concrete approach and an explicit construction that elucidates how the properties of the exponential family and the dually flat geometry may be extended to the constant curvature case. In this paper, a careful exposition of the λ-deformation framework is provided from the perspective of λ-duality, namely the λ-deformation of Legendre duality.

The rest of the paper is organized as follows. In Section 2, we review the standard S-model of information geometry with a focus on the dually flat geometry, based on convex duality and Bregman divergence, of the exponential and mixture families. The section closes with a preview of λ-deformation by introducing a suite of four deformation functions, as two pairs of mutually inverse functions: $\log_{\lambda }$ versus $\exp_{\lambda }$ and $\kappa_{\lambda }$ versus $\gamma_{\lambda }$, with the first pair deforming log and exp and the second pair deforming the identity function. In Section 3, we describe the λ-duality, which deforms the standard convex duality. In particular, we compare λ-duality and standard Legendre duality and show their relations to each other upon a change of parameterization. In Section 4, we define the λ-gradient and then the λ-logarithmic divergence and study the constant curvature information geometry the latter induces. In Section 5, we relate λ-divergence to Rényi entropy by introducing the λ-exponential and λ-mixture family. The two expressions of the λ-exponential family under divisive and subtractive normalization correspond to, respectively, Rényi deformation and Tsallis deformation. Section 6 concludes with a comparison of λ-deformation with the standard dually flat (Hessian) framework.

## 2. The Standard Model of Information Geometry

### 2.1. The Standard Model

We begin by recalling the standard framework (referred to as the S-model) of parametric information geometry [1,20]. Let M be a finite-dimensional differentiable manifold with dimension *d* and $\theta =(\theta^{1},\ldots ,\theta^{d})$ be a local coordinate system. The most important case is where M is a manifold of parametric probability density functions. However, the idea of deforming Legendre duality to λ-duality and hence dually flat (Hessian) manifolds to manifolds of constant curvature discussed in Section 3 and Section 4 is entirely general and does not rely on M being a manifold of probability density functions.

Let (X,μ) be a measure space, where μ is called the reference (or dominating) measure. Let $\Theta \subset R^{d}$ be an open domain. A parametric family of density functions is a mapping θ∈Θ↦p(·|θ), where each p(·|θ) is a probability density function with respect to μ, i.e., $\int_{X}p\left(\zeta |\theta \right)d\mu \left(\zeta \right)=1$. We assume that the family is sufficient regular such that all analytical operations (such as differentiation under the integral sign) can be performed as needed.

While a dualistic structure $\left(M,g,\nabla ,\nabla^{*}\right)$ can be defined abstractly, in practice, it is often constructed by a *divergence*, namely a smooth, non-negative function D[·||·] on M×M such that $D[p||p^{\prime }]=0$ only if $p=p^{\prime }$ and the (0,2)-tensor g it induces on M (see (2) below) is positive definite. Intuitively, $D[p||p^{\prime }]$ defines a notion of “asymmetric distance” between points *p* and $p^{\prime }$ of M. When M is a manifold of density functions, a prominent example is the *Kullback–Leibler (KL) divergence* (relative entropy) given by:

$$H[p||p^{\prime }]=\int p\log\frac{p}{p^{\prime }}d\mu .$$

When dealing with parametric probability families, *p* and $p^{\prime }$ are replaced by p(·|θ) and $p(\cdot |\theta^{\prime })$, then we denote $D[p||p^{\prime }]$ as $D(\theta ,\theta^{\prime })$ with an abuse of notation, that is:

$$D[p\left(\cdot |\theta \right)||p\left(\cdot |\theta^{\prime }\right)]\equiv D\left(\theta ,\theta^{\prime }\right),$$

and similarly for *H* as well—the notation of $\left[p||p^{\prime }\right]$ in the divergence for probability density functions emphasizes the non-symmetricity in *p*, $p^{\prime }$; see [1].

Eguchi [21] showed that any divergence function (called a “contrast function” there) induces a dualistic structure $\left(M,g,\nabla ,\nabla^{*}\right)$. In local coordinates, given $D(\theta ,\theta^{\prime })$, the components $g_{ij}$ of the metric g are given by:

(2) $$g_{ij}\left(\theta \right)=-{\left.\frac{\partial^{2}}{\partial \theta^{i}\partial \theta^{\prime j}}D\left(\theta ,\theta^{\prime }\right)\right|}_{\theta =\theta^{\prime }}={\left.\frac{\partial^{2}}{\partial \theta^{i}\partial \theta^{j}}D\left(\theta ,\theta^{\prime }\right)\right|}_{\theta =\theta^{\prime }},$$

and the Christoffel symbols of the conjugate connections ∇ and $\nabla^{*}$ are given respectively by:

(3) $$\Gamma_{ij,k}\left(\theta \right)=-{\left.\frac{\partial^{3}}{\partial \theta^{i}\partial \theta^{j}\partial \theta^{\prime k}}D\left(\theta ,\theta^{\prime }\right)\right|}_{\theta^{\prime }=\theta },\;\Gamma_{ij,k}^{*}\left(\theta \right)=-{\left.\frac{\partial^{3}}{\partial \theta^{\prime i}\partial \theta^{\prime j}\partial \theta^{k}}D\left(\theta ,\theta^{\prime }\right)\right|}_{\theta =\theta^{\prime }}.$$

Conversely, given any dualistic structure $\left(M,g,\nabla ,\nabla^{*}\right)$, there exists a divergence *D* that induces it, but this *D* is not unique in general [22]. Thus, the standard model S is completely encoded by the choice of a divergence function.

### 2.2. Dually Flat Geometry

The most important example of a dualistic structure is the *dually flat geometry,* which is induced by a Bregman divergence [23]. Let M be prescribed with an affine coordinate chart θ∈Θ on an open convex set $\Theta \subset R^{d}$. Let ϕ:Θ→R be a differentiable convex function; specifically, we assumed that ϕ is $C^{2}$ and its Hessian $D^{2}\phi$ is strictly positive definite. The *Bregman divergence* of ϕ is defined by:

$$B_{\phi }\left(\theta ,\theta^{\prime }\right)=\phi \left(\theta \right)-\phi \left(\theta^{\prime }\right)-D\phi \left(\theta^{\prime }\right)\cdot \left(\theta -\theta^{\prime }\right),\;\theta ,\theta^{\prime }\in \Theta ,$$

where $D\phi \left(\theta \right)={\left(\frac{\partial }{\partial \theta^{1}}\phi \left(\theta \right),\ldots ,\frac{\partial }{\partial \theta^{d}}\phi \left(\theta \right)\right)}^{⊤}$ is the Euclidean gradient and a·b denotes the standard dot product. We call θ∈Θ the *primal coordinates*, and η=Dϕ(θ) the *dual coordinates*, where the inverse of Dϕ is given by $\theta =D\phi^{*}\left(\eta \right)$. Here, the Legendre conjugate $\phi^{*}$ (or convex conjugate) of ϕ is defined by:

(4) $$\phi^{*}\left(\eta \right)=\underset{\theta }{\sup}\left(\theta \cdot \eta -\phi (\theta )\right).$$

Then, the components $g_{ij}$ of the Riemannian metric g, under the respective local coordinate system, are given by:

(5) $$g_{ij}\left(\theta \right)=\frac{\partial^{2}}{\partial \theta^{i}\partial \theta^{j}}\phi \left(\theta \right),\;g_{ij}\left(\eta \right)=\frac{\partial^{2}}{\partial \eta^{i}\partial \eta^{j}}\phi^{*}\left(\eta \right).$$

In particular, g is a *Hessian metric* with potential ϕ (resp. $\phi^{*}$) under θ (resp. η). Furthermore, the Christoffel symbols of ∇ and $\nabla^{*}$ are given respectively by:

(6) $$\Gamma_{ij,k}\left(\theta \right)=0,\;\Gamma_{ij,k}^{*}\left(\eta \right)=0.$$

From (6), we see that the Riemann curvature tensors of both ∇ and $\nabla^{*}$ vanish. Thus, we call this a *dually flat* geometry. Furthermore, a ∇-geodesic (resp. $\nabla^{*}$-geodesic) is a constant velocity straight line under the θ (resp. η) coordinate system.

Moreover, the θ and η coordinates are *biorthogonal* in the sense that:

(7) $$g\left(\frac{\partial }{\partial \theta^{i}},\frac{\partial }{\partial \eta^{j}}\right)=\delta_{ij},$$

and the Bregman divergence takes the forms of:

$$B_{\phi }\left(\theta ,\theta^{\prime }\left(\eta^{\prime }\right)\right)=A_{\phi }\left(\theta ,\eta^{\prime }\right)=A_{\phi^{*}}\left(\eta^{\prime },\theta \right)=B_{\phi^{*}}\left(\eta^{\prime },\eta \left(\theta \right)\right)$$

with η=Dϕ(θ) and $\theta^{\prime }=D\phi^{*}\left(\eta^{\prime }\right)$, where *A* is called the *canonical divergence*:

(8) $$A_{\phi }\left(\theta ,\eta^{\prime }\right)=\phi \left(\theta \right)+\phi^{*}\left(\eta^{\prime }\right)-\theta \cdot \eta^{\prime }=A_{\phi^{*}}\left(\eta^{\prime },\theta \right).$$

Following [24,25], we call the equality between two expressions of *B* and the equality between two expressions of *A* in (8) *reference–representation biduality*. In [26], the identity (8) was used to motivate a family of *Fenchel–Young losses* in the context of regularized prediction in machine learning. Last but not least, the Bregman divergence satisfies the *generalized Pythagorean theorem*: given points *P*, *Q*, and *R*, we have the equality:

$$B_{\phi }\left(\theta_{Q},\theta_{P}\right)+B_{\phi }\left(\theta_{R},\theta_{Q}\right)=B_{\phi }\left(\theta_{R},\theta_{P}\right)$$

if and only if the ∇-geodesic between *Q* and *R* and the $\nabla^{*}$-geodesic between *Q* and *P* meet g-orthogonally at *Q*. As we will see in Section 4, all the properties above have natural generalizations under our λ-framework. We stress that the dually flat geometry depends crucially on classical convex (or Legendre) duality, as seen from (4) and (8).

### 2.3. Exponential and Mixture Families

The dually flat Hessian geometry arises naturally in the *exponential* and *mixture families* of probability densities. Given a reference measure μ on a state space X, an *exponential family* is a parameterized probability density $p^{\left(e\right)}\left(\cdot |\theta \right)$ of the form:

(9) $$p^{\left(e\right)}\left(\zeta |\theta \right)=e^{\theta \cdot F(\zeta )-\phi (\theta )},$$

where $\theta =\left(\theta^{1},\ldots ,\theta^{d}\right)\in \Theta \subseteq R^{d}$ and $F\left(\zeta \right)=(F_{1}\left(\zeta \right),\ldots ,F_{d}\left(\zeta \right))$ is a vector of *sufficient statistics*. In (9), the *cumulant generating function* ϕ, defined by:

$$\phi \left(\theta \right)=\log\int e^{\theta \cdot F}d\mu ,$$

enforces the normalization $\int p^{\left(e\right)}d\mu =1$. The exponential family generalizes the Boltzmann–Gibbs distribution in statistical physics, where $Z\left(\theta \right)=e^{\phi (\theta )}$ is called the partition function.

The information geometry of the exponential family begins with the observation that ϕ is convex. Then, ϕ defines a Bregman divergence $B_{\phi }$ giving rise to a dually flat structure. It can be shown that the Bregman divergence is a KL-divergence:

$$B_{\phi }\left(\theta ,\theta^{\prime }\right)=H[p^{\left(e\right)}\left(\cdot |\theta^{\prime }\right)\left|\right|p^{\left(e\right)}\left(\cdot |\theta \right)].$$

The induced Riemannian metric g, the Fisher–Rao metric given by (in matrix components $g_{ij}$), becomes a Hessian metric $D^{2}\phi$:

$$g_{ij}\left(\theta \right)=\int \left(\frac{\partial }{\partial \theta^{i}}\log p^{\left(e\right)}\left(\zeta |\theta \right)\right)\left(\frac{\partial }{\partial \theta^{j}}\log p^{\left(e\right)}\left(\zeta |\theta \right)\right)p^{\left(e\right)}\left(\zeta |\theta \right)d\mu =\frac{\partial^{2}}{\partial \theta^{i}\partial \theta^{j}}\phi \left(\theta \right).$$

Equivalently, g(θ) is the covariance matrix of the sufficient statistics *F*:

$$g_{ij}\left(\theta \right)=\int p^{\left(e\right)}\left(\zeta |\theta \right)\left(F_{i}\left(\zeta \right)-\int p^{\left(e\right)}\left(\zeta |\theta \right)F_{i}\left(\zeta \right)\right)\left(F_{j}\left(\zeta \right)-\int p^{\left(e\right)}\left(\zeta |\theta \right)F_{j}\left(\zeta \right)\right)d\mu .$$

Furthermore, the dual coordinate η=Dϕ(θ) is the *expectation coordinates* given by:

$$\eta =\int p^{\left(e\right)}\left(\zeta |\theta \right)F\left(\zeta \right)d\mu ,$$

and the dual potential function $\phi^{*}$ is, as a function of η, the negative Shannon entropy:

$$\phi^{*}\left(\eta \right)=-H\left[p^{\left(e\right)}\left(\cdot |\theta \right)\right]=\int p^{\left(e\right)}\left(\zeta |\theta \right)\log p^{\left(e\right)}\left(\zeta |\theta \right)d\mu .$$

A theoretical justification for the exponential family is that it maximizes the Shannon entropy under the constraints of its expected value of the vector of random functions F(·).

The *mixture family* is another probability family that is very useful in both theory and applications. Let $P_{0}\left(\zeta \right),P_{1}\left(\zeta \right),\ldots ,P_{d}\left(\zeta \right)$ be a set of affinely independent probability densities with respect to the same dominating measure μ. Given mixture parameters $\eta_{i}>0$ for i=0,⋯,d with $\sum_{i=0}^{d}\eta_{i}=1$, the mixture family $p^{\left(m\right)}\left(\cdot |\eta \right)$ is defined by:

$$p^{\left(m\right)}\left(\zeta |\eta \right)=\sum_{i=0}^{d}\eta_{i}P_{i}\left(\zeta \right)=P_{0}\left(\zeta \right)+\sum_{i=1}^{d}\eta_{i}\left(P_{i}\left(\zeta \right)-P_{0}\left(\zeta \right)\right),$$

where $\left(\eta_{1},\ldots ,\eta_{d}\right)$ may be taken as the independent parameters. It can be shown that the negative Shannon entropy:

$$\psi \left(\eta \right)=-H\left[p^{\left(m\right)}\left(\cdot |\eta \right)\right]=\int p^{\left(m\right)}\left(\zeta |\eta \right)\log p^{\left(m\right)}\left(\zeta |\eta \right)d\mu$$

of a mixture family is convex in η. Using ψ as the potential function, we have:

$$B_{\psi }\left(\eta ,\eta^{\prime }\right)=H[p^{\left(m\right)}\left(\cdot |\eta \right)\left|\right|p^{\left(m\right)}\left(\cdot |\right|\eta^{\prime })],$$

which is again a KL-divergence and induces a dually flat geometry. In summary, the exponential and mixture families are both dually flat when the geometry is induced by the KL-divergence.

For completeness, we note that the convex conjugate of ψ(η) is:

$$\psi^{*}\left(\theta \right)=-\int P_{0}\left(\zeta \right)\;\log p^{\left(m\right)}\left(\zeta |\eta \right)\;d\mu ,$$

with conjugate parameters θ=Dψ given by:

$$\theta^{i}=\int \left(P_{i}\left(\zeta \right)-P_{0}\left(\zeta \right)\right)\;\log p^{\left(m\right)}\left(\zeta |\eta \right)\;d\mu .$$

### 2.4. Deforming *exp* and *log*

The exponential function used in the exponential family:

$$p^{\left(e\right)}\left(\zeta |\theta \right)=\exp\left\{\theta \cdot F\left(\zeta \right)-\phi \left(\theta \right)\right\}=\frac{\;e^{\theta \cdot F(\zeta )}}{Z(\theta )}$$

allows the cumulant generating function ϕ(θ) (also called the potential function) and the partition function Z(θ) to be linked by the simple relation ϕ=logZ. The equivalence of using ϕ as subtractive normalization and *Z* as divisive normalization of the same exponential family $\int p^{\left(e\right)}\left(\zeta |\theta \right)d\mu =1$ is due to the elementary, but crucial property exp(x+y)=exp(x)exp(y) of the exponential function. Using a functional form other than exp (exponential function) or log (logarithm function) is referred to as *deformation* in information geometric (statistical and information theoretic) contexts, and the resulting probability families are called “deformed” families. Typically, this is performed by regarding log, or equivalently exp, as special cases of some parametric class of functions that include them as special members.

More generally, the exponential/logarithmic function can be considered within a non-parametric function space that includes exp or log as a special member. Several approaches can be found in the literature, including the ϕ-deformed exponential approach by Naudts [5,27,28], the conjugate (ρ,τ)-embedding approach by the first author [8,25,29], and the *U*-model by Eguchi [7,30]. The ϕ-model and *U*-model are both one-function models, while the (ρ,τ)-model uses two free functions. It eventually became clear in the 2018 paper [6] by Naudts and the first author that (i) the ϕ- and *U*-model turned out to be equivalent; (ii) they are special cases of the (ρ,τ)-model upon a particular fixing of the “gauge freedom”; (iii) the corresponding (ρ,τ)-geometry of the manifold of the ϕ-exponential family can have different appearances (gauges freedom), such as a Hessian geometry (under one type of gauge selection) and a conformal Hessian geometry (under another type of gauge selection). The work [6] unified the intermediary results in [10,11,31] and provided a general deformation framework that preserves the rigid interlocking of: (i) the functional form of entropy, cross-entropy, and relative entropy (divergence); (ii) the functional form of the deformed probability family with the corresponding normalization and potential and the duality between the natural and expectation parameterizations; (iii) the expressions of the Riemannian metric (Fisher–Rao metric in general and Hessian metric in particular) and of the conjugate connections. Some of these concepts have their correspondence in nonparametric probability families as well [32,33,34].

Although the (ρ,τ)-model may admit a conformal Hessian metric (more rigorously stated: the ϕ-exponential family with the (ρ,τ)-metric under a certain gauge will lead to conformal Hessian geometry), the dual connections are not projectively flat (as the geometry studied by [12]). As a result, while the connections are not flat (torsion-free, but not curvature-free), they are not in general of the constant-curvature-type either. Therefore they are “too general” and do not generate the space of constant curvatures.

### 2.5. Highlights of λ-Deformation

Here enters λ-deformation as a middle ground [17]. The λ-deformation theory absorbs the *q*-deformation model of Tsallis and the $F^{\left(\pm \alpha \right)}$ model of Wong [12] in deforming the exponential family and unifies the subtractive and divisive normalization—this is an occasion where subtractive and divisive normalizations are still linked by a simple reparameterization of the probability family.

Let us introduce some notations. Consider the following deformed logarithm and exponential functions (note the slight difference to the $\log_{q}$ notation used by Tsallis, in the way how the subscript indicates the deformation parameter):

$$\log_{\lambda }\left(t\right)=\frac{1}{\lambda }\left(t^{\lambda }-1\right),\;\exp_{\lambda }\left(t\right)={\left(1+\lambda t\right)}^{1/\lambda }.$$

More precisely, we define $\exp_{\lambda }:R\to \left[0,\infty \right]$ (where λ∈R,λ≠0) by:

$$\exp_{\lambda }\left(t\right)={\left[1+\lambda \;t\right]}_{+}^{1/\lambda },$$

where ${\left[a\right]}_{+}=\max\left\{a,0\right\}$. In our analysis, we assumed implicitly that 1+λt>0, which is shown to hold for λ-duality, so the subscript + can be omitted. Furthermore, $\frac{d}{dt}\exp_{\lambda }\left(t\right)={\left[\exp_{\lambda }\left(t\right)\right]}^{1-\lambda }$, so $\exp_{\lambda }\left(\cdot \right)$ is convex if and only if λ<1. For this reason, we restricted λ to this range as in [9,28]. Below, we also took logt=−∞ whenever t≤0. Note that our notation differs slightly from Tsallis’ indexing of the deformed logarithm and exponential functions; see Section 5.

Next, we construct another pair of inverse functions $\kappa_{\lambda },\gamma_{\lambda }$ by:

$$\kappa_{\lambda }=\log\circ \exp_{\lambda },\;\gamma_{\lambda }=\log_{\lambda }\circ \exp,$$

where ∘ denotes function composition. Explicitly, they are:

(10) $$\kappa_{\lambda }\left(t\right)=\frac{1}{\lambda }\log\left(1+\lambda t\right),\;\gamma_{\lambda }\left(t\right)=\frac{1}{\lambda }\left(e^{\lambda t}-1\right).$$

This suite of four functions, namely $\exp_{\lambda },\log_{\lambda }$ as an inverse pair and $\kappa_{\lambda },\gamma_{\lambda }$ as another inverse pair, is called *λ-deformation* and used in the discussions of λ-convexity, λ-conjugation, and λ-duality. Regular exponential and logarithmic functions are recovered when λ→0, whence both $\kappa_{\lambda }$ and $\gamma_{\lambda }$ reduce to the identity function.

Using these four functions, Wong and Zhang [17] developed the λ-deformation framework to solve the problem of relating the exponential family under subtractive normalization:

$$p^{\left(\lambda \right)}\left(\zeta |\theta \right)=\exp_{\lambda }\left(\theta \cdot F\left(\zeta \right)-\phi_{\lambda }\left(\theta \right)\right)$$

to that under divisive normalization:

$$p^{\left(\lambda \right)}\left(\zeta |\vartheta \right)=\exp_{\lambda }\left(\vartheta \cdot F\left(\zeta \right)\right)e^{-\varphi_{\lambda }\left(\vartheta \right)}.$$

There, the same λ-deformed exponential family can be expressed by two parameterizations θ and ϑ linked through:

$$\theta =\vartheta e^{-\lambda \varphi_{\lambda }\left(\vartheta \right)}\;\;⟺\;\;\vartheta =\frac{\theta }{1-\lambda \phi_{\lambda }\left(\theta \right)}\;\;,$$

while the normalization functions $\phi_{\lambda }$ and $\varphi_{\lambda }$ (with different domains) are linked through:

$$\phi_{\lambda }\left(\theta \right)=\gamma_{-\lambda }\left(\varphi_{\lambda }\left(\vartheta \right)\right)\;\;⟺\;\;\varphi_{\lambda }\left(\vartheta \right)=\kappa_{-\lambda }\left(\phi_{\lambda }\left(\theta \right)\right)\;\;.$$

The λ-deformation framework led to a unified way of looking at the Tsallis entropy (related to the subtractive denormalization) and Rényi entropy (related to the divisive normalization), as well as generating new insights into the distinction between the exponential and mixture families through the lens of deformation theory. To understand this deformation better, we describe the underlying mathematical framework of λ-deformation.

## 3. Deforming the Legendre Duality: λ-Duality

In this section, we describe the λ-duality and a its link to the standard Legendre duality. We start by defining the notions of λ-conjugate and λ-convexity/λ-concavity, then draw a parallel to the regular Legendre duality. We proceed to establish a formal correspondence between the λ-duality and classical convex duality, including the associated notions of the λ-gradient, λ-logarithmic divergence, etc. Some of the derivations are illustrative, yet heuristic—a rigorous analysis in the spirit of Rockafellar [35] is yet to be performed in future research.

### 3.1. Legendre Duality and Bregman Divergence Reviewed

Recall from (4) that the *convex conjugate* of a function *f* on $R^{d}$ is defined by:

(11) $$f^{*}\left(u\right)=\underset{x}{\sup}\left(x\cdot u-f(x)\right),\;u\in R^{d}.$$

It can be proven that:

(i) $f^{*}$ is convex;
(ii) ${\left({\left(f^{*}\right)}^{*}\right)}^{*}=f^{*}$;
(iii) ${\left(f^{*}\right)}^{*}=f$ if *f* is convex and lower semicontinuous.

When *f* is further differentiable, then the Legendre transformation:

u=Df(x),

which can be motivated by the first-order condition in (11), defines a “dual variable” *u*, satisfying the Fenchel identity:

$$f\left(x\right)+f^{*}\left(u\right)=x\cdot u.$$

We have $x=Df^{*}\left(u\right)$, provided the second derivative or $D^{2}f$ is positive definite. The function *f* also defines a Bregman divergence $B_{f}$ given by:

(12) $$B_{f}\left(x,x^{\prime }\right)=f\left(x\right)-f\left(x^{\prime }\right)-Df\left(x^{\prime }\right)\cdot \left(x-x^{\prime }\right)\geq 0.$$

The Bregman divergence satisfies the *reference–representation biduality* [24,25] in the sense that:

$$B_{f}\left(x,x^{\prime }\right)=B_{f^{*}}\left(u^{\prime },u\right)$$

where $u=Df\left(x\right),u^{\prime }=Df\left(x^{\prime }\right).$ Note that when *f* is convex and differentiable, the non-negativity of the Bregman divergence encodes the fact that for any $x,x^{\prime }$:

$$f\left(x\right)-f\left(x^{\prime }\right)\geq Df\left(x^{\prime }\right)\cdot \left(x-x^{\prime }\right).$$

### 3.2. λ-Deformation of Legendre Duality

The main idea behind the λ-deformation of the Legendre duality (“λ-duality”) is to replace the term x·u in (11) by a monotone transformation of x·u. Given a parameter λ∈R\{0}, later revealed to be the *curvature parameter* of the information geometric characterization, we replace the term x·u by:

(13) $$\kappa_{\lambda }\left(x\cdot u\right)=\frac{1}{\lambda }\log\left(1+\lambda x\cdot u\right),$$

where $\kappa_{\lambda }\left(t\right)$ and its inverse $\gamma_{\lambda }\left(t\right)$ are given by (10). With this in mind we give the following definition.

**Definition** **1** (λ-conjugation)**.** *Let $\Omega ,\Omega^{\prime }\subset R^{d}$. Given a function f:Ω→R, we define its λ-conjugate $f^{\left(\lambda \right)}$ by:*

(14) $$f^{\left(\lambda \right)}\left(u\right)=\underset{x\in \Omega }{\sup}\left(\kappa_{\lambda }\left(x\cdot u\right)-f\left(x\right)\right),\;u\in \Omega^{\prime }.$$

Generalized convex dualities have been heavily used in optimal transport theory [36,37] to characterize the optimal transport plans; in this context, it is called the *c*-duality where *c* is the cost function of the transport problem. A major novelty of our framework is that the functional form of $\kappa_{\lambda }$ (and of $\gamma_{\lambda }$) leads to explicit formulas, which are not available in the general case. We remark that this is closely related to the fact that the associated information geometry has constant curvature λ.

It turns out that the λ-conjugation defined by (14) corresponds to an appropriately generalized notion of convexity or concavity, through the aid of the function $\gamma_{\lambda }$ given by (10). Henceforth, we let λ∈R\{0} be a fixed constant.

**Definition** **2** (λ-exponential convexity and concavity)**.** *Let $\Omega \subset R^{d}$ be an open convex set. A function f:Ω→R is said to be λ-exponentially convex (“λ-convex”), or λ-exponentially concave (“λ-concave”), if:*

$$G_{\lambda ,f}^{}\left(x\right)=\left(\gamma_{\lambda }\circ f\right)\left(x\right)=\frac{1}{\lambda }\left(e^{\lambda f(x)}-1\right)$$

*is convex, or concave, on *Ω*. When f is $C^{2}$, we have equivalently that f is λ-convex, or λ-concave, if the Hessian of $G_{\lambda ,f}^{}\equiv \gamma_{\lambda }\circ f$ is positive definite, or negative definite.*

Note that the additive term −1/λ in the above definition of $G_{\lambda ,f}^{}\left(x\right)=\frac{1}{\lambda }\left(e^{\lambda f(x)}-1\right)$ is not necessary; it is included so that $\lim_{\lambda \to 0}G_{\lambda ,f}^{}\left(x\right)=f\left(x\right)$, meaning that in the limiting case of zero-convexity is just ordinary convexity.

It is easily shown that, for λ>0 a fixed positive number,

(i) *f* is λ-convex if and only if −f is (−λ)-concave;
(ii) *f* is λ-concave if and only if −f is (−λ)-convex.

**Proposition** **1.** *Given any f:Ω→R, we define variable $\tilde{x}$, which has range $\tilde{\Omega }\subset R^{d}$, and function $g:\tilde{\Omega }\to R$ by:*

(15) $$\begin{array}{ccc} \tilde{x} & = & x\;e^{-\lambda \;f(x)}=x\;\left(1-\lambda G_{-\lambda ,f}\left(x\right)\right), \end{array}$$

(16) $$\begin{array}{ccc} g(\tilde{x}) & = & \frac{1}{-\lambda }\left(e^{-\lambda f(x)}-1\right)=\gamma_{-\lambda }\left(f\left(x\right)\right)=G_{-\lambda ,f}^{}\left(x\right). \end{array}$$

Then, the convex (Legendre) conjugate $g^{*}$ of the function g:

$$g^{*}\left(u\right)=\underset{\tilde{x}\in \tilde{\Omega }}{\sup}\left(\tilde{x}\cdot u-g\left(\tilde{x}\right)\right)$$

*is related to the λ-conjugate $f^{\left(\lambda \right)}$ of the function f via:*

(17) $$g^{*}\left(u\right)=\frac{1}{\lambda }\left(e^{\lambda f^{\left(\lambda \right)}\left(u\right)}-1\right)=\gamma_{\lambda }\left(f^{\left(\lambda \right)}\left(u\right)\right)=G_{\lambda ,f^{\left(\lambda \right)}}^{}\left(u\right)\;.$$

**Proof.**  We first prove the following identities:

$$\begin{array}{ccc} \left(1+\lambda x\cdot u\right)e^{-\lambda f(x)} & = & e^{-\lambda f(x)}+\lambda \;e^{-\lambda f(x)}x\cdot u\\ & = & \left(1-\lambda \;g\left(\tilde{x}\right)\right)+\lambda \;\tilde{x}\cdot u\\ & = & 1+\lambda \;(\tilde{x}\cdot u-g\left(\tilde{x}\right)) \end{array}$$

where, going from the first to the second line, we used (15) and the fact:

$$1-\lambda g\left(\tilde{x}\right)=e^{-\lambda f(x)},$$

which is a re-write of the definition of *g* given by (16).

With the above identity, we can proceed to prove this proposition. For $u\in \Omega^{\prime }$, we have:

$$\begin{array}{ccc} f^{\left(\lambda \right)}\left(u\right) & = & \underset{x\in \Omega }{\sup}\left(\frac{1}{\lambda }\log\left(1+\lambda \left(x\cdot u\right)\right)-f\left(x\right)\right)\\ & = & \underset{\tilde{x}\in \tilde{\Omega }}{\sup}\frac{1}{\lambda }\log\left(1+\lambda \;(\tilde{x}\cdot u-g\left(\tilde{x}\right))\right)\\ & = & \frac{1}{\lambda }\log\left(1+\lambda \;\underset{\tilde{x}\in \tilde{\Omega }}{\sup}\left(\tilde{x}\cdot u-g\left(\tilde{x}\right)\right)\right)\\ & = & \frac{1}{\lambda }\log\left(1+\lambda g^{*}\left(u\right)\right)\\ & = & \kappa_{\lambda }\left(g^{*}\left(u\right)\right). \end{array}$$

Recasting the above relation yields (17). □

Recall that from convex analysis, $g^{*}$ is always a convex function regardless of whether *g* is convex (by the property of Legendre conjugation). The expression of $g^{*}\left(u\right)=\gamma_{\lambda }\left(f^{\left(\lambda \right)}\left(u\right)\right)$ in (17) therefore implies that $f^{\left(\lambda \right)}$ is λ-convex, by the definition of λ-convexity.

**Corollary** **1.** *For any f:Ω→R, its λ-conjugate $f^{\left(\lambda \right)}\left(u\right)$ as defined by *(14)* is a λ-convex function of u on $\Omega^{\prime }$ (note $\Omega^{\prime }$ may not necessarily be convex).*

**Proof.**  We can also give a direct proof (essentially reversing the steps of the proof of Proposition 1).

$$\begin{array}{ccc} g^{*}\left(u\right) & = & \underset{\tilde{x}\in \tilde{\Omega }}{\sup}\left(\tilde{x}\cdot u-g\left(\tilde{x}\right)\right)\\ & = & \underset{x\in \Omega }{\sup}\left(e^{-\lambda f(x)}\left(x\cdot u\right)-\frac{1}{-\lambda }\left(e^{-\lambda f(x)}-1\right)\right)\\ & = & \underset{x\in \Omega }{\sup}\frac{1}{\lambda }\left(\left(1+\lambda x\cdot u\right)e^{-\lambda f(x)}\right)-\frac{1}{\lambda }\\ & = & \frac{1}{\lambda }\left(\underset{x\in \Omega }{\sup}e^{\;\log(1+\lambda x\cdot u)-\lambda f(x)}-1\right)\\ & = & \frac{1}{\lambda }\left(e^{\;\sup_{x\in \Omega }\left(\log\left(1+\lambda x\cdot u\right)-\lambda f\left(x\right)\right)}-1\right)\\ & = & \frac{1}{\lambda }\left(e^{\lambda f^{\left(\lambda \right)}\left(u\right)}-1\right)\\ & = & \gamma_{\lambda }\left(f^{\left(\lambda \right)}\left(u\right)\right). \end{array}$$

□

Corollary 1 is the extension of the claim that for any *f*, the standard Legendre conjugate $f^{*}$ as given by (11) is always a convex function. Because of this, we can prove, in analogy to the standard Legendre conjugation ∗, the following relations:

(i) ${\left({\left(f^{\left(\lambda \right)}\right)}^{\left(\lambda \right)}\right)}^{\left(\lambda \right)}=f^{\left(\lambda \right)}$ for any *f*.
(ii) ${\left(f^{\left(\lambda \right)}\right)}^{\left(\lambda \right)}=f$ if *f* is λ-convex.

### 3.3. Relations between the λ-Duality and Legendre Duality

We proceed to establish a formal relationship between the λ-duality and the ordinary Legendre duality, by relating the λ-conjugation of a λ-convex function *f*, denoted by $f^{\left(\lambda \right)}$, to the standard Legendre conjugation of a function (denoted by ∗).

We continue the analysis performed in Proposition 1. Taking λ-conjugation for a second time,

$$\begin{array}{ccc} {\left(f^{\left(\lambda \right)}\right)}^{\left(\lambda \right)}\left(x\right) & = & \underset{u\in \Omega^{\prime }}{\sup}\frac{1}{\lambda }\left(\log(1+\lambda (x\cdot u))\right)-f^{\left(\lambda \right)}\left(u\right)\\ & = & \underset{\tilde{u}\in \Omega^{\prime }}{\sup}\frac{1}{\lambda }\left(\log(1+\lambda \;\left(x\cdot \tilde{u}-\tilde{g}\left(\tilde{u}\right)\right))\right)\\ & = & \frac{1}{\lambda }\log\left(1+\lambda \;\underset{\tilde{u}\in \Omega^{\prime }}{\sup}\left(x\cdot \tilde{u}-\tilde{g}\left(\tilde{u}\right)\right)\right)\\ & = & \frac{1}{\lambda }\log\left(1+\lambda {\tilde{g}}^{*}\left(x\right)\right)\\ & = & \kappa_{\lambda }\left({\tilde{g}}^{*}\left(x\right)\right). \end{array}$$

Here, the variable $\tilde{u}$ is defined by:

$$\tilde{u}=u\;e^{-\lambda \;f^{\left(\lambda \right)}\left(u\right)},$$

and the function $\tilde{g}$ by:

(18) $$\tilde{g}\left(\tilde{u}\right)=\gamma_{-\lambda }\left(f^{\left(\lambda \right)}\left(u\right)\right)=G_{-\lambda ,f^{\left(\lambda \right)}}^{}\left(u\right).$$

In the event when *f* is λ-convex, then ${\left(f^{\left(\lambda \right)}\right)}^{\lambda }=f$. Therefore:

$${\tilde{g}}^{*}\left(x\right)=\gamma_{\lambda }\left(f\left(x\right)\right)=G_{\lambda ,f}^{}\left(x\right).$$

Therefore, $\tilde{g}\left(\tilde{u}\right)={\left(G_{\lambda ,f}^{}\right)}^{*}\left(\tilde{u}\right)$. That is, the function $\tilde{g}$ is just the (regular) Legendre conjugation ${}^{*}$ of the function $G_{\lambda ,f}^{}\left(x\right)$. In $\tilde{u}$ parameterization, the $\tilde{g}$ function has the expression of (18) with $\tilde{u}$ and *u* related by (20). This parallels the fact that $g\left(\tilde{x}\right)={\left(G_{\lambda ,f^{\left(\lambda \right)}}^{}\right)}^{*}\left(\tilde{x}\right)$, and in $\tilde{x}$ parameterization, the *g* function has the expression of (16) with $\tilde{x}$ and *x* related by (19).

Summarizing the above, we have:

**Theorem** **1** (Connecting λ-duality to Legendre duality)**.** *Let f be a λ-convex function and $f^{\left(\lambda \right)}$ be its λ-conjugate. Denote two functions g and $\tilde{g}$:*

$$\begin{array}{ccc} g(\tilde{x}) & = & G_{-\lambda ,f}^{}\left(x\right)=\gamma_{-\lambda }\left(f\left(x\right)\right),\\ \tilde{g}\left(\tilde{u}\right) & = & G_{-\lambda ,f^{\left(\lambda \right)}}^{}\left(u\right)=\gamma_{-\lambda }\left(f^{\left(\lambda \right)}\left(u\right)\right), \end{array}$$

*where the two variables $\tilde{x}$ and $\tilde{u}$ are given by:*

(19) $$\begin{array}{ccc} & & \tilde{x}=x\;e^{-\lambda \;f(x)}⟺x=\frac{\tilde{x}}{1-\lambda g(\tilde{x})}, \end{array}$$

(20) $$\begin{array}{ccc} & & \tilde{u}=u\;e^{-\lambda \;f^{\left(\lambda \right)}\left(u\right)}⟺u=\frac{\tilde{u}}{1-\lambda \tilde{g}\left(\tilde{u}\right)}. \end{array}$$

*Then, the following statements are equivalent:*

*(i)*  *The (x,u) variables satisfy the λ-duality of a pair of λ-convex functions $\left(f,f^{\left(\lambda \right)}\right)$:*
  (21) $$\kappa_{\lambda }\left(x\cdot u\right)=f\left(x\right)+f^{\left(\lambda \right)}\left(u\right);$$
*(ii)*  The $\left(\tilde{x},u\right)$ variables satisfy the Legendre duality of a pair of convex functions $\left(g,g^{*}\right)$:
  (22) $$\tilde{x}\cdot u=g\left(\tilde{x}\right)+g^{*}\left(u\right)$$
  *with:*
  $$g^{*}\left(u\right)=G_{\lambda ,f^{\left(\lambda \right)}}^{}\left(u\right)=\gamma_{\lambda }\left(f^{\left(\lambda \right)}\left(u\right)\right);$$
*(iii)*  The $\left(x,\tilde{u}\right)$ variables satisfy the Legendre duality of a pair of convex functions $\left(\tilde{g},{\tilde{g}}^{*}\right)$:
  (23) $$x\cdot \tilde{u}={\tilde{g}}^{*}\left(x\right)+\tilde{g}\left(\tilde{u}\right)$$
  *with:*
  $${\tilde{g}}^{*}\left(x\right)=G_{\lambda ,f}^{}\left(x\right)=\gamma_{\lambda }\left(f\left(x\right)\right).$$

**Proof.**  To prove the equivalence of (21) and (22), we re-write the latter as:

$$e^{-\lambda f(x)}x\cdot u=\gamma_{-\lambda }\left(f\left(x\right)\right)+\gamma_{\lambda }\left(f^{\left(\lambda \right)}\left(u\right)\right)=\frac{1}{\lambda }\left(e^{\lambda f^{\left(\lambda \right)}\left(u\right)}-e^{-\lambda f(x)}\right),$$

where we inserted the following relations:

$$g\left(\tilde{x}\right)=\gamma_{-\lambda }\left(f\left(x\right)\right),\;g^{*}\left(u\right)=\gamma_{\lambda }\left(f^{\left(\lambda \right)}\left(u\right)\right)$$

and replaced $\tilde{x}$ by *x* using (19). Multiplying $e^{\lambda f(x)}$ on both sides, we obtain:

(24) $$x\cdot u=\frac{1}{\lambda }\left(e^{\lambda (f^{\left(\lambda \right)}\left(u\right)+f\left(x\right))}-1\right)=\gamma_{\lambda }\left(f^{\left(\lambda \right)}\left(u\right)+f\left(x\right)\right).$$

Noting ${\left(\gamma_{\lambda }\right)}^{-1}=\kappa_{\lambda }$ verifies (21). To prove the equivalence of (21) and (23), we rely on an analogous identity:

$$\left(1+\lambda x\cdot u\right)e^{-\lambda f^{\left(\lambda \right)}\left(u\right)}=1+\lambda \;\left(x\cdot \tilde{u}-\tilde{g}\left(\tilde{u}\right)\right),$$

where:

$$\tilde{u}=u\;e^{-\lambda \;f^{\left(\lambda \right)}\left(u\right)},\;\;\;\;\;\;\;\;\tilde{g}\left(\tilde{u}\right)=\gamma_{-\lambda }\left(f^{\left(\lambda \right)}\left(u\right)\right).$$

We have, after multiplying $e^{\lambda f^{\left(\lambda \right)}\left(u\right)}$ on both sides of (24),

$$x\cdot ue^{-\lambda f^{\left(\lambda \right)}\left(u\right)}=\frac{1}{\lambda }\left(e^{\lambda f(x)}-e^{-\lambda f^{\left(\lambda \right)}\left(u\right)}\right)=\gamma_{\lambda }\left(f\left(x\right)\right)+\gamma_{-\lambda }\left(f^{\left(\lambda \right)}\left(u\right)\right)={\tilde{g}}^{*}\left(x\right)+\tilde{g}\left(\tilde{u}\right),$$

where the last step used:

$${\tilde{g}}^{*}\left(x\right)=\gamma_{\lambda }\left({\left(f^{\left(\lambda \right)}\right)}^{\left(\lambda \right)}\left(x\right)\right)\;,\;\;\;\;\;\;\;\;\tilde{g}\left(\tilde{u}\right)=\gamma_{-\lambda }\left(f^{\left(\lambda \right)}\left(u\right)\right).$$

Noting ${\left(f^{\left(\lambda \right)}\right)}^{\left(\lambda \right)}=f$ due to *f* assumed to be λ-convex, then (23) follows. □

We see that the functions $\gamma_{\lambda }$ and $\gamma_{-\lambda }$ serve as link functions from the $\left(f,f^{\left(\lambda \right)}\right)$-pair of the λ-deformed Legendre conjugation to the $\left(g,g^{*}\right)$-pair and the $\left(\tilde{g},{\tilde{g}}^{*}\right)$-pair of the regular Legendre conjugation.

## 4. λ-Logarithmic Divergence and Its Dualistic Geometry

In this section, we study the λ-deformation of the Bregman (canonical) divergence function and the resulting dualistic geometry (Riemannian metric and dual connections), which correspond to the λ-duality. This involves first establishing the λ-deformation to the gradient operation (so-called λ-gradient), which then leads to the so-called λ-logarithmic divergence function as deformation to the Bregman divergence. Finally, we show that the resulting Riemannian metric is a conformal Hessian metric, while the resulting dual connections are projectively flat (with constant curvature). The conformal and projective factor is parameterized by λ, which gives the curvature of the constant curvature space.

### 4.1. λ-Gradient

**Definition** **3** (λ-gradient)**.** *For x∈Ω, define the λ-gradient $D^{\left(\lambda \right)}f$ by:*

(25) $$D^{\left(\lambda \right)}f\left(x\right)=\frac{1}{1-\lambda Df(x)\cdot x}Df\left(x\right).$$

The work of [17] (Theorem 2.2) showed the above formula for deforming the gradient of a function motivated by the λ-duality setting. For mathematical convenience, it is proven under some regularity conditions; a full generalization along the lines of [35] is a natural direction for further research.

**Theorem** **2** (λ-gradient for λ-duality)**.** *Let λ≠0, and let f be a λ-exponentially convex function that is $C^{2}$ on some open convex set $\Omega \subset R^{d}$, such that (a) $D^{2}G_{\lambda ,f}^{}$ is strictly positive definite and (b) 1−λDf(x)·x>0 on *Ω*. Then we have*

*(i)*  *$D^{\left(\lambda \right)}f$ is a $C^{1}$-diffeomorphism from *Ω* to its range $\Omega^{\prime }$.*
*(ii)*  *Denote $u=D^{\left(\lambda \right)}f\left(x\right)$. We have 1+λx·u>0, and the following identity holds:*
  $$f\left(x\right)+f^{\left(\lambda \right)}\left(u\right)=\frac{1}{\lambda }\log\left(1+\lambda x\cdot u\right)\equiv \kappa_{\lambda }\left(x\cdot u\right).$$
*(iii)*  *Furthermore, $x=D^{\left(\lambda \right)}f^{\left(\lambda \right)}\left(u\right)$.*

Note that the λ-gradient $D^{\left(\lambda \right)}f$ differs from the regular gradient Df by a scalar multiplication. The duality between *x* and *u* under the λ-duality is mediated by a dual variable $u=D^{\left(\lambda \right)}f\left(x\right)$, which plays an important role in what follows.

Let:

(a) $u=D^{\left(\lambda \right)}f\left(x\right)$ denote the λ-conjugate variable corresponding to *x* with respect to f(x);
(b) $\hat{u}=Dg\left(\tilde{x}\right)$ be the Legendre conjugate variable corresponding to $\tilde{x}$ with respect to $g(\tilde{x})$;
(c) $x=D^{\left(\lambda \right)}f^{\left(\lambda \right)}\left(u\right)$ denote the λ-conjugate variable corresponding to *u* with respect to $f^{\left(\lambda \right)}\left(u\right)$;
(d) $\hat{x}=D\tilde{g}\left(\tilde{u}\right)$ be the Legendre conjugate variable corresponding to $\tilde{u}$ with respect to $\tilde{g}\left(\tilde{u}\right)$.

Is there a simple relationship between them? The following proposition says $u\left(x\right)=\hat{u}\left(\tilde{x}\right)$, where $\tilde{x}$ and *x* are linked by (19), and $x\left(u\right)=\hat{x}\left(\tilde{u}\right)$, where $\tilde{u}$ and *u* are linked by (20).

**Proposition** **2.** *We have:*

$$u=D_{x}^{\left(\lambda \right)}f\left(x\right)=D_{\tilde{x}}^{}g\left(\tilde{x}\right)\;,\;\;\;\;\;\;\;\;x=D_{u}^{\left(\lambda \right)}f^{\left(\lambda \right)}\left(u\right)=D_{\tilde{u}}^{}\tilde{g}\left(\tilde{u}\right)$$

*Here, we add the subscript to D to emphasize the argument with respect to which the derivative is taken.*

**Proof.**  We use matrix notations where the gradient is regarded as a column vector. Applying the multivariate chain rule to (17), we have:

$${\left(D_{\tilde{x}}g\left(\tilde{x}\right)\right)}^{⊤}=e^{-\lambda f(x)}{\left(D_{x}f\left(x\right)\right)}^{⊤}\frac{\partial x}{\partial \tilde{x}}\left(\tilde{x}\right),$$

where $\frac{\partial x}{\partial \tilde{x}}\left(\tilde{x}\right)$ is the Jacobian of the transformation $\tilde{x}\mapsto x$ and ${\left(\cdot \right)}^{⊤}$ denotes the transpose, For two vectors *x* and *y*, their outer product is denoted by $x\;y^{⊤}$, which is a rank-one square matrix with the (i,j)-entry $x^{i}y^{j}$.

From (15), we have:

$$\frac{\partial \tilde{x}}{\partial x}\left(x\right)=e^{-\lambda f(x)}\left(I-\lambda \;x\;{\left(D_{x}f\left(x\right)\right)}^{⊤}\right).$$

Since $1-\lambda D_{x}f\left(x\right)\cdot x>0$ by assumption, we can invert the Jacobian by the Sherman–Morrison formula (see [12], Proposition 4) to obtain:

$$\frac{\partial x}{\partial \tilde{x}}\left(\tilde{x}\right)=e^{\lambda f(x)}\left(I+\frac{\lambda \;x\;{\left(D_{x}f\left(x\right)\right)}^{⊤}}{1-\lambda D_{x}f\left(x\right)\cdot x}\right).$$

Plugging this into the above, we have:

$${\left(D_{\tilde{x}}g\left(\tilde{x}\right)\right)}^{⊤}=\frac{{\left(D_{x}f\left(x\right)\right)}^{⊤}}{1-\lambda D_{x}f\left(x\right)\cdot x}\;\;.$$

Using (25) to relate $D_{x}^{\left(\lambda \right)}f\left(x\right)$ to $D_{x}f\left(x\right)$, the first relation involving $D_{\tilde{x}}g\left(\tilde{x}\right)$ is proven. The proof of the second relation in this proposition is analogous. □

Just as ordinary convexity leads to the notion of Bregman divergence (12), the notion of λ-exponential convexity leads to a generalization that we call the *λ-logarithmic divergence*. Henceforth, we let f:Ω→R be a λ-exponentially convex function on an open convex domain $\Omega \subset R^{d}$, and we assumed that the regularity conditions in Theorem 2 hold.

### 4.2. λ-Logarithmic Divergence

By the definition of the λ-convexity, we have that $G_{\lambda ,f}^{}\left(x\right)=\gamma_{\lambda }\left(f\left(x\right)\right)$ is convex on Ω. By the ordinary convexity of $G_{\lambda ,f}^{}$, we have:

$$G_{\lambda ,f}^{}\left(x\right)-G_{\lambda ,f}^{}\left(x^{\prime }\right)\geq DG_{\lambda ,f}^{}\left(x^{\prime }\right)\cdot \left(x-x^{\prime }\right),\;x,x^{\prime }\in \Omega .$$

In terms of *f*, we have, after some manipulations,

$$\gamma_{\lambda }\left(f\left(x\right)-f\left(x^{\prime }\right)\right)\geq Df\left(x^{\prime }\right)\cdot \left(x-x^{\prime }\right).$$

Since $\gamma_{\lambda }$ is increasing, we have:

$$f\left(x\right)-f\left(x^{\prime }\right)\geq {\left(\gamma_{\lambda }\right)}^{-1}\left(Df\left(x^{\prime }\right)\cdot \left(x-x^{\prime }\right)\right)=\kappa_{\lambda }\left(Df\left(x^{\prime }\right)\cdot \left(x-x^{\prime }\right)\right).$$

This motivates the following definition.

**Definition** **4** (λ-logarithmic divergence)**.** *We define the λ-logarithmic divergence of f by:*

(26) $$\begin{array}{cc} L_{\lambda ,f}\left(x,x^{\prime }\right) & =f\left(x\right)-f\left(x^{\prime }\right)-\kappa_{\lambda }\left(Df\left(x^{\prime }\right)\cdot \left(x-x^{\prime }\right)\right)\\ \; & =f\left(x\right)-f\left(x^{\prime }\right)-\frac{1}{\lambda }\log\left(1+\lambda Df\left(x^{\prime }\right)\cdot \left(x-x^{\prime }\right)\right),\;x,x^{\prime }\in \Omega . \end{array}$$

**Figure 1:**
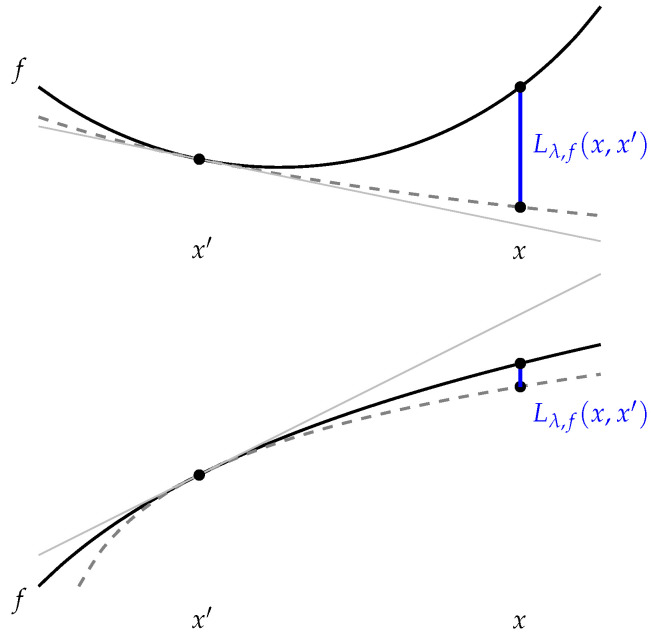
Illustration of the λ-logarithmic divergence. Top: λ=−1 and $f\left(x\right)=\sqrt{x(10-x)}$. Bottom: λ=1 and f(x)=2logx. In both cases, $x^{\prime }=4$ and x=8, and we plot the function on the interval (2,9). Note that the first-order logarithmic approximation (dashed grey curve) supports the graph of *f* from below.

See Figure 1 for a graphical illustration. We note that the logarithmic correction in (26) corresponds to a *logarithmic first-order approximation*, based at $x^{\prime }$, which is possible due to the λ-exponential convexity of *f*. We also note that when λ>0, it is possible that $L_{\lambda ,f}\left(x,x^{\prime }\right)=\infty$. Nevertheless, $L_{\lambda ,f}\left(x,x^{\prime }\right)$ is finite when *x* and $x^{\prime }$ are sufficiently close. Formally, letting λ→0 in (26) recovers the Bregman divergence.

### 4.3. λ-Logarithmic Divergence in Different Forms

We now prove a lemma about the relationship of the variables x,u and gradients or λ-gradients of *f* or $f^{\left(\lambda \right)}$. We assumed, for convenience, that 1+λx·u>0 for all $x\in \Omega ,u\in \Omega^{\prime }$.

**Lemma** **1.** *Given $u=D_{x}^{\left(\lambda \right)}f\left(x\right)$ or equivalently $x=D_{u}^{\left(\lambda \right)}f^{\left(\lambda \right)}\left(u\right)$, for arbitrary $x^{\prime },u^{\prime }$ (such that the expressions are well defined), we have the following identities:*

(27) $$\begin{array}{ccc} \kappa_{\lambda }\left(u\cdot x^{\prime }\right)-\kappa_{\lambda }\left(u\cdot x\right) & = & \kappa_{\lambda }\left(u\cdot \left(x^{\prime }-x\right)\;{\left(\Pi_{\lambda }\right)}^{-1}\right), \end{array}$$

(28) $$\begin{array}{ccc} \kappa_{\lambda }\left(u^{\prime }\cdot x\right)-\kappa_{\lambda }\left(u\cdot x\right) & = & \kappa_{\lambda }\left(\left(u^{\prime }-u\right)\cdot x\;{\left(\Pi_{\lambda }\right)}^{-1}\right). \end{array}$$

*where $\Pi_{\lambda }$ is a multiplicative factor (function of x or u) given by:*

$$\Pi_{\lambda }\equiv 1+\lambda x\cdot u=\frac{1}{1-\lambda \;Df(x)\cdot x}=\frac{1}{1-\lambda Df^{\left(\lambda \right)}\left(u\right)\cdot u}.$$

**Proof.**  Since $u=D_{x}^{\left(\lambda \right)}f\left(x\right)$, substituting (25), we have:

$$u\cdot x=\frac{Df(x)\cdot x}{1-\lambda \;Df(x)\cdot x}$$

and:

$$1+\lambda \;u\cdot x=\frac{1}{1-\lambda \;Df(x)\cdot x}$$

so:

$$1+\lambda \;u\cdot x^{\prime }=\frac{1+\lambda \;Df\left(x\right)\cdot \left(x^{\prime }-x\right)}{1-\lambda \;Df(x)\cdot x}=\left(1+\lambda u\cdot x\right)\left(1+\lambda \;Df\left(x\right)\cdot \left(x^{\prime }-x\right)\right).$$

Taking the logarithm and rearranging, we obtain (27).

On the other hand, because:

$$x=D_{u}^{\left(\lambda \right)}f^{\left(\lambda \right)}\left(u\right)=\frac{Df^{\left(\lambda \right)}\left(u\right)}{1-\lambda Df^{\left(\lambda \right)}\left(u\right)\cdot u}\;\;,$$

we also have:

$$1+\lambda x\cdot u=\frac{1}{1-\lambda Df^{\left(\lambda \right)}\left(u\right)\cdot u}\;\;.$$

The proof of (28) is similar. □

In this above lemma, $x^{\prime }$ and $u^{\prime }$ are arbitrary; it is interesting that a modified form of “linearity” holds even though $\kappa_{\lambda }$ is itself nonlinear. As a consequence, we have an alternative expression for $L_{\lambda ,f}\left(x,x^{\prime }\right)$.

**Proposition** **3.** *$L_{\lambda ,f}\left(x,x^{\prime }\right)$ defined by *(26)* can also be written as:*

$$L_{\lambda ,f}\left(x,x^{\prime }\right)=f\left(x\right)-f\left(x^{\prime }\right)-\kappa_{\lambda }\left(x\cdot u^{\prime }\right)+\kappa_{\lambda }\left(x^{\prime }\cdot u^{\prime }\right).$$

*where $u^{\prime }=D^{\left(\lambda \right)}f\left(x^{\prime }\right)$.*

Of course, we may express the λ-logarithmic divergence using the conjugate variables $u,u^{\prime }$ as well. Indeed, we have the analogous reference–representation biduality (see [24,25]) that is characteristic of Bregman divergence and canonical divergence for dually flat spaces, that is (8). See [38] for the reference–representation biduality of a general *c-divergence* (which includes both the Bregman and logarithmic divergences) based on optimal transport.

**Theorem** **3.** *The λ-logarithmic divergence satisfies the reference–representation biduality, namely:*

$$L_{\lambda ,f^{\left(\lambda \right)}}\left(u^{\prime },u\right)=L_{\lambda ,f}\left(x,x^{\prime }\right),$$

*where $u=D^{\left(\lambda \right)}f\left(x\right)$ and $u^{\prime }=D^{\left(\lambda \right)}f\left(x^{\prime }\right)$. Moreover, define the λ-deformed canonical divergence $A_{\lambda ,f}$ by:*

$$A_{\lambda ,f}\left(x,u^{\prime }\right)=f\left(x\right)+f^{\left(\lambda \right)}\left(u^{\prime }\right)-\frac{1}{\lambda }\log\left(1+\lambda x\cdot u^{\prime }\right)=A_{\lambda ,f^{\left(\lambda \right)}}\left(u^{\prime },x\right).$$

*We have:*

$$L_{\lambda ,f}\left(x,x^{\prime }\right)=A_{\lambda ,f}\left(x,u^{\prime }\right)=A_{\lambda ,f^{\left(\lambda \right)}}\left(u^{\prime },x\right)=L_{\lambda ,f^{\left(\lambda \right)}}\left(u^{\prime },u\right).$$

Proposition 3 also allows us to derive our next theorem (Theorem 4) linking λ-logarithmic divergence and Bregman divergence (also see [19] for a discussion of conformal divergence in the affine immersion setting).

**Theorem** **4.** The canonical forms of the λ-logarithmic divergence $A_{\lambda ,f}$ and $A_{\lambda ,f^{\left(\lambda \right)}}$ are related to the canonical forms of the Bregman divergence $A_{g^{*}}$ and $A_{\tilde{g}}$ via a conformal transformation and the non-linear link function $\kappa_{-\lambda }$:

$$\begin{array}{ccc} & & A_{\lambda ,f^{\left(\lambda \right)}}\left(u^{\prime },x\right)\;=\;\kappa_{-\lambda }\left(e^{-\lambda f^{\left(\lambda \right)}\left(u^{\prime }\right)}A_{g^{*}}\left(u^{\prime },\tilde{x}\right)\right),\\ & = & A_{\lambda ,f}\left(x,u^{\prime }\right)\;=\;\kappa_{-\lambda }\left(e^{-\lambda f(x)}A_{{\tilde{g}}^{*}}\left(x,\tilde{u^{\prime }}\right)\right). \end{array}$$

**Proof.**  $$\begin{array}{cc} A_{\lambda ,f^{\left(\lambda \right)}}\left(u^{\prime },x\right) & =f^{\left(\lambda \right)}\left(u^{\prime }\right)+f\left(x\right)-\frac{1}{\lambda }\log\left(1+\lambda u^{\prime }\cdot x\right)\\ \;\; & =f^{\left(\lambda \right)}\left(u^{\prime }\right)-\frac{1}{\lambda }\log\left(\left(1+\lambda u^{\prime }\cdot x\right)e^{-\lambda f(x)}\right)\\ \;\; & =\frac{1}{\lambda }\log\left(1+\lambda g^{*}\left(u^{\prime }\right)\right)-\frac{1}{\lambda }\log\left(e^{-\lambda f(x)}+\lambda u^{\prime }\cdot \left(xe^{-\lambda f(x)}\right)\right)\\ \;\; & =\frac{1}{\lambda }\log\left(1+\lambda g^{*}\left(u^{\prime }\right)\right)-\frac{1}{\lambda }\log\left(1-\lambda g\left(\tilde{x}\right)+\lambda u^{\prime }\cdot \tilde{x}\right)\\ \;\; & =-\frac{1}{\lambda }\log\left(\frac{1+\lambda (u^{\prime }\cdot \tilde{x}-g\left(\tilde{x}\right))}{1+\lambda g^{*}\left(u^{\prime }\right)}\right)\\ \;\; & =-\frac{1}{\lambda }\log\left(1+\lambda \frac{u^{\prime }\cdot \tilde{x}-g\left(\tilde{x}\right)-g^{*}\left(u^{\prime }\right)}{1+\lambda g^{*}\left(u^{\prime }\right)}\right)\\ \;\; & =-\frac{1}{\lambda }\log\left(1-\lambda \frac{A_{g^{*}}\left(u^{\prime },\tilde{x}\right)}{1+\lambda g^{*}\left(u^{\prime }\right)}\right)\\ \;\; & =-\frac{1}{\lambda }\log\left(1-\lambda \left(e^{-\lambda f^{\left(\lambda \right)}\left(u^{\prime }\right)}\;A_{g^{*}}\left(u^{\prime },\tilde{x}\right)\right)\right)\\ \;\; & =\kappa_{-\lambda }\left(e^{-\lambda f^{\left(\lambda \right)}\left(u^{\prime }\right)}\;A_{g^{*}}\left(u^{\prime },\tilde{x}\right)\right). \end{array}$$

The proof of the second line of Theorem 4 is similar. We have $A_{\lambda ,f^{\left(\lambda \right)}}\left(u^{\prime },x\right)=A_{\lambda ,f}\left(x,u^{\prime }\right)$ from Theorem 3. □

### 4.4. Dualistic Geometry of λ-Logarithmic Divergence

Regard x∈Ω as the primal (global) coordinate system of a manifold M. As described in Section 2.1, we may use the λ-logarithmic divergence $L_{\lambda ,f}$ of *f* to construct a dualistic structure $\left(M,g,\nabla ,\nabla^{*}\right)$. In this subsection, we provide explicit expressions of the corresponding coefficients and state some key geometric consequences.

We begin with the Riemannian metric.

**Theorem** **5.** *The Riemannian metric g induced from $L_{\lambda ,f}\left(x,x^{\prime }\right)$ is given in primal coordinate x by:*

(29) $$g\left(x\right)=D^{2}f\left(x\right)+\lambda \left(Df\left(x\right)\right){\left(Df\left(x\right)\right)}^{⊤}=e^{-\lambda f(x)}D^{2}G_{\lambda ,f}^{}\left(x\right).$$

**Proof.**  According to (2), we perform direct differentiation of (26):

$$g_{ij}\left(x\right)={\left.\frac{\partial^{2}}{\partial x^{i}\partial x^{j}}L_{\lambda ,f}\left(x,x^{\prime }\right)\right|}_{x=x^{\prime }}$$

and obtain the expression of (29). □

By symmetry, under the dual coordinate system $u=D^{\left(\lambda \right)}f\left(x\right)$, we have:

$$g\left(u\right)=D^{2}f^{\left(\lambda \right)}\left(u\right)+\lambda \left(Df^{\left(\lambda \right)}\left(u\right)\right){\left(Df^{\left(\lambda \right)}\left(u\right)\right)}^{⊤}.$$

From the first equality in (29), we see that g is a *rank-one correction* of the Hessian matrix $D^{2}f\left(x\right)$. From the second equality, we see that g is in fact a *conformal Hessian metric*, i.e., it has the form $g\left(x\right)=e^{-\lambda f(x)}g_{0}\left(x\right)$, where $g_{0}\left(x\right)=D^{2}G_{\lambda ,f}^{}\left(x\right)$ is the Hessian metric induced by the convex function $G_{\lambda ,f}^{}\left(x\right)=\frac{1}{\lambda }\left(e^{\lambda f(x)}-1\right)$. This conclusion is entirely anticipated from Theorem 4.

To compute the Christoffel symbols of the primal and dual connections, we need an expression of the inverse of the Riemannian metric g(x) as a matrix. This is provided by the following proposition.

**Proposition** **4.** *The metric g can be expressed as:*

(30) $$g\left(x\right)=\frac{1}{\Pi_{\lambda }\left(x\right)}\left(I_{d}-\frac{\lambda }{\Pi_{\lambda }\left(x\right)}ux^{⊤}\right)\frac{\partial u}{\partial x}\left(x\right),$$

*where $\frac{\partial u}{\partial x}$ is the Jacobian matrix of the coordinate transformation x↦u and $I_{d}$ is the d×d identity matrix with Kronecker $\delta_{ij}$ as its entries. Here:*

$$\Pi_{\lambda }\left(x\right)=1+\lambda x\cdot u=\frac{1}{1-\lambda \;Df(x)\cdot x}$$

*and $\Pi_{\lambda }\left(x\right)>0$ for x∈Ω and $u=D^{\left(\lambda \right)}f\left(x\right)$, due to Part (ii) of Theorem 2.*

*Moreover, the inverse of g(x) can be expressed as:*

(31) $${\left(g\left(x\right)\right)}^{-1}=\Pi_{\lambda }\left(x\right)\frac{\partial x}{\partial u}\left(u\right)\left(I_{d}+\lambda ux^{⊤}\right).$$

**Proof.**  Using the λ-logarithmic divergence represented as the generalized canonical divergence $A_{\lambda ,f}$ (26), we apply (2) to obtain:

$$\begin{array}{cc} g_{ij}\left(x\right) & =-{\left.\frac{\partial^{2}}{\partial x^{i}\partial x^{\prime j}}L_{\lambda ,f}\left(x,x^{\prime }\right)\right|}_{x=x^{\prime }}\\ \; & =-{\left.\frac{\partial^{2}}{\partial x^{i}\partial x^{\prime j}}\left\{f\left(x\right)+f^{\left(\lambda \right)}\left(u^{\prime }\right)-\frac{1}{\lambda }\log\left(1+\lambda x\cdot u^{\prime }\right)\right\}\right|}_{x=x^{\prime }}\\ \; & =\frac{1}{\Pi_{\lambda }\left(x\right)}\left\{\frac{\partial u^{i}}{\partial x^{j}}-\frac{\lambda }{\Pi_{\lambda }\left(x\right)}u^{i}\sum_{k=1}^{d}x^{k}\frac{\partial u^{k}}{\partial x^{j}}\right\}. \end{array}$$

Expressing the above expression using matrix notations gives (30). Formula (31) follows by inverting (30) using the Sherman–Morrison formula. □

Under the dualistic structure induced by a λ-logarithmic divergence, the primal and dual coordinate vector fields are no longer biorthogonal in the sense of (7). Nevertheless, we have the following generalization. Again, we write $\Pi_{\lambda }\left(x\right)=1+\lambda x\cdot u$.

**Corollary** **2.** *The inner product of the coordinate vector fields $\frac{\partial }{\partial x^{i}}$ and $\frac{\partial }{\partial u^{j}}$ is given by a λ-deformed “biorthogonality” relation:*

$$g\left(\frac{\partial }{\partial x^{i}},\frac{\partial }{\partial u^{j}}\right)=\frac{1}{\Pi_{\lambda }\left(x\right)}\delta_{ij}-\frac{\lambda }{\Pi_{\lambda }{\left(x\right)}^{2}}x^{j}u^{i}.$$

**Proof.**  Write $\frac{\partial }{\partial u^{j}}=\sum_{m=1}^{d}\frac{\partial x^{m}}{\partial u^{j}}\frac{\partial }{\partial x^{m}}$. Then:

$$g\left(\frac{\partial }{\partial x^{i}},\frac{\partial }{\partial u^{j}}\right)=\sum_{m=1}^{d}\frac{\partial x^{m}}{\partial u^{j}}\;g\left(\frac{\partial }{\partial x^{i}},\frac{\partial }{\partial x^{m}}\right).$$

Simplifying the expression using (30) gives the result. For details, see ([12], Proposition 8). □

**Theorem** **6.** *The Christoffel symbols of the primal connection *∇* are given by:*

$$\Gamma_{ij,k}\left(x\right)=-\frac{\lambda }{\Pi_{\lambda }{\left(x\right)}^{2}}\left(u^{j}\frac{\partial u^{i}}{\partial x^{k}}+u^{i}\frac{\partial u^{j}}{\partial x^{k}}\right)+\frac{2\lambda^{2}}{\Pi_{\lambda }{\left(x\right)}^{3}}\sum_{\ell =1}^{d}u^{i}u^{j}x^{\ell }\frac{\partial u^{\ell }}{\partial x^{k}},$$

*where $\Pi_{\lambda }\left(x\right)=1+\lambda x\cdot u$ as in Proposition 4. Furthermore, let $\Gamma_{ij}^{k}=\sum_{\ell =1}^{d}\Gamma_{ij,\ell }g^{\ell k}$ be the Christoffel symbol of the second kind, then:*

(32) $$\Gamma_{ij}^{k}\left(x\right)=\frac{-\lambda }{\Pi_{\lambda }\left(x\right)}\left(u^{i}\delta_{j}^{k}+u^{j}\delta_{i}^{k}\right)=-\lambda \left(\frac{\partial f}{\partial x^{i}}\left(x\right)\delta_{j}^{k}+\frac{\partial f}{\partial x^{j}}\left(x\right)\delta_{i}^{k}\right),$$

*where δ is the Kronecker delta.*

Similarly, under the dual coordinate system u, the Christoffel symbol (of the second kind) of the dual connection $\nabla^{*}$ is given by:

(33) $$\Gamma_{ij}^{*k}\left(u\right)=-\lambda \left(\frac{\partial f^{\left(\lambda \right)}}{\partial u^{i}}\left(u\right)\delta_{j}^{k}+\frac{\partial f^{\left(\lambda \right)}}{\partial u^{j}}\left(u\right)\delta_{i}^{k}\right).$$

**Proof.**  This is a straightforward computation using (3) and Proposition 4. The details, which are a minor modification of the proof of ([12], Proposition 5), are omitted. □

Although the curvatures of ∇ and $\nabla^{*}$ are nonzero, it can be shown that ∇ and $\nabla^{*}$ are both *projectively flat*, i.e., each of them is projectively equivalent to a flat connection. Specifically, any ∇-geodesic (resp. $\nabla^{*}$-geodesic) is a time-reparameterized straight line under the *x* (resp. *u*) coordinate system.

**Theorem** **7.** The sectional curvatures of *∇* and $\nabla^{*}$ with respect to g are both equal to λ.

**Proof.**  See ([12], Theorem 15). □

Using the dual projective flatness and Corollary 2, Reference ([12], Theorem 16) showed that the λ-logarithmic divergence satisfies a generalized Pythagorean theorem, which generalizes the property of Bregman divergence outlined in Section 2.2.

**Theorem** **8** (Generalized Pythagorean theorem)**.** *Let P,Q,R∈M. Then:*

$$L_{\lambda ,f}\left(x_{Q},x_{P}\right)+L_{\lambda ,f}\left(x_{R},x_{Q}\right)=L_{\lambda ,f}\left(x_{R},x_{P}\right)$$

if and only if the *∇*-geodesic between Q and R and the $\nabla^{*}$-geodesic between Q and P meet g-orthogonally at Q.

To summarize, the dually flat geometry becomes a dually *projectively flat* geometry with *constant* sectional curvature λ, and the Hessian metric becomes a *conformal* Hessian metric. Nevertheless, the primal and dual geodesics are still straight lines (up to time reparametrizations), and the generalized Pythagorean theorem holds.

We say that the above λ-deformation framework is “canonical” because the statistical manifold $\left(M,g,\nabla ,\nabla^{*}\right)$, with a conformal Hessian metric $g_{ij}$ given by (29) and a pair of dual projectively flat affine connections $\Gamma_{ij}^{k},\Gamma_{ij}^{*k}$ given by (32) and (33), is the *only* statistical structure with constant curvature ([12], Theorem 15). Moreover, given such a statistical manifold, one can construct locally a λ-logarithmic divergence, which induces the given geometry.

## 5. Linking λ-Deformation to Rényi Entropy and Divergence

### 5.1. Relation between Tsallis’ and Rényi’s Deformation Expressions

Recall that Tsallis [39], in the context of statistical physics, introduced the generalized entropy:

$$H_{\lambda }^{\mathrm{Tsallis}}\left[p\right]=\int p\log_{\lambda }\left(\frac{1}{p}\right)\;d\mu =\frac{1}{\lambda }\left(\int {\left(p\left(\zeta \right)\right)}^{\lambda }-1\right)\;d\mu ;$$

note that we use λ here in place of q=1−λ as in [40].

Tsallis entropy is related to Rényi entropy [41], defined as:

$$H_{\lambda }^{\mathrm{Rényi}}\left[p\right]:=\frac{1}{\lambda }\log\left(\int p^{1-\lambda }\left(\zeta \right)\;d\mu \right),$$

through a monotonic transformation:

$$H_{\lambda }^{\mathrm{Tsallis}}\left[p\right]=\frac{1}{\lambda }\left(e^{\lambda H_{\lambda }^{\mathrm{Rényi}}\left[p\right]}-1\right).$$

In our current notation,

$$H_{\lambda }^{\mathrm{Tsallis}}\left[p\right]=\gamma_{\lambda }\left(H_{\lambda }^{\mathrm{Rényi}}\left[p\right]\right)⟺H_{\lambda }^{\mathrm{Rényi}}\left[p\right]=\kappa_{\lambda }\left(H_{\lambda }^{\mathrm{Tsallis}}\left[p\right]\right).$$

Rényi divergence (with Rényi index 1−λ) is defined by:

$$H_{\lambda }^{\mathrm{Rényi}}\left[p|\right|p^{\prime }]=\frac{-1}{\lambda }\log\int {\left(p\left(\zeta \right)\right)}^{1-\lambda }{\left(p^{\prime }\left(\zeta \right)\right)}^{\lambda }d\mu .$$

Rényi divergence is *additive*: given two product measures $p_{1}⊗p_{2}$ and $p_{1}^{\prime }⊗p_{2}^{\prime }$, we have:

$$H_{\lambda }^{\mathrm{Rényi}}[p_{1}⊗p_{2}\left|\right|p_{1}^{\prime }⊗p_{2}^{\prime }]=H_{\lambda }^{\mathrm{Rényi}}[p_{1}\left|\right|p_{1}^{\prime }]+H_{\lambda }^{\mathrm{Rényi}}[p_{2}\left|\right|p_{2}^{\prime }].$$

Because Tsallis entropy is not additive, this has been used as an argument for favoring Rényi entropy as a physical concept over Tsallis entropy; see [28] (Section 9.3) and [42].

### 5.2. λ-Exponential Family

Under the λ-deformation, there is an intrinsic link between the subtractive and divisive normalizations of the λ-deformed exponential family. Starting with the observation:

$$e^{\kappa_{\lambda }\left(t\right)}={\left(1+\lambda t\right)}^{1/\lambda }=\exp_{\lambda }\left(t\right),$$

we investigate the identity:

$${\left(1+\lambda \vartheta \cdot F\left(\zeta \right)\right)}^{1/\lambda }\;e^{-\varphi_{\lambda }\left(\vartheta \right)}={\left(1+\lambda \left(\theta \cdot F\left(\zeta \right)-\phi_{\lambda }\left(\theta \right)\right)\right)}^{1/\lambda }.$$

Taking the λ-th power and equating both sides, we obtain the conditions for the above identity to hold:

$$\begin{array}{ccc} \theta =\vartheta e^{-\lambda \varphi_{\lambda }\left(\vartheta \right)}\;\; & ⟺ & \;\;\vartheta =\frac{\theta }{1-\lambda \phi_{\lambda }\left(\theta \right)}\;\;,\\ \phi_{\lambda }\left(\theta \right)=\frac{1}{-\lambda }\left(e^{-\lambda \varphi_{\lambda }\left(\vartheta \right)}-1\right)\;\; & ⟺ & \;\;\varphi_{\lambda }\left(\vartheta \right)=-\frac{1}{\lambda }\log\left(1-\lambda \phi_{\lambda }\left(\theta \right)\right). \end{array}$$

This fact led us to define a λ-exponential family that can be normalized both subtractively and divisively: the former denoted by p(ζ|θ) and the latter denoted by p(ζ|ϑ).

**Proposition** **5** (Reparameterization equivalence)**.** *Let λ≠0. With respect to a given reference measure μ and a fixed vector of random functions $F\left(\zeta \right)=(F_{1}\left(\zeta \right),\ldots ,F_{d}\left(\zeta \right))$, the λ-exponential family is given by $p^{\left(\lambda \right)}\left(\zeta |\theta \right)$ under subtractive normalization and by $p^{\left(\lambda \right)}\left(\zeta |\vartheta \right)$ under divisive normalization; they are reparametrizations of each other:*

(34) $$p^{\left(\lambda \right)}\left(\zeta |\theta \right)=\exp_{\lambda }\left(\theta \cdot F\left(\zeta \right)-\phi_{\lambda }\left(\theta \right)\right)=\exp_{\lambda }\left(\vartheta \cdot F\left(\zeta \right)\right)e^{-\varphi_{\lambda }\left(\vartheta \right)}=p^{\left(\lambda \right)}\left(\zeta |\vartheta \right).$$

Here, the function $\phi_{\lambda }\left(\theta \right)$ is called *subtractive* λ-potential and used for *subtractive* normalization, while $\varphi_{\lambda }\left(\vartheta \right)$ is called *divisive* λ-potential and used for *divisive* normalization. Note that $\phi_{\lambda }$ and $\varphi_{\lambda }$ may not have same domains. They satisfy:

$$\phi_{\lambda }\left(\theta \right)=\gamma_{-\lambda }\left(\varphi_{\lambda }\left(\vartheta \right)\right)\;\;⟺\;\;\varphi_{\lambda }\left(\vartheta \right)=\kappa_{-\lambda }\left(\phi_{\lambda }\left(\theta \right)\right)\;\;,$$

where:

$$\kappa_{-\lambda }\left(t\right)=-\frac{1}{\lambda }\log\left(1-\lambda t\right)\;\;,\;\;\;\;\;\;\;\;\;\;\;\gamma_{-\lambda }\left(t\right)=\frac{1}{\lambda }\left(1-e^{-\lambda t}\right)\;\;.$$

Note again that we use ϑ for the divisive normalization setting and distinguish it from θ for the subtractive normalization setting. For later convenience, we also note:

$$e^{-\lambda \varphi_{\lambda }\left(\vartheta \right)}=1-\lambda \phi_{\lambda }\left(\theta \right).$$

#### 5.2.1. Under Subtractive Normalization

The deformed exponential family takes the form:

(35) $$p\left(\zeta |\theta \right)=\exp_{\lambda }\left(\theta \cdot F\left(\zeta \right)-\phi_{\lambda }\left(\theta \right)\right)$$

where $\theta \cdot F\left(\zeta \right)=\sum_{i=1}^{d}\theta^{i}F_{i}\left(\zeta \right)$, and the subtractive λ-potential $\phi_{\lambda }\left(\theta \right)$ is specified by the normalization:

$$1=\int p\left(\zeta |\theta \right)\;d\mu =\int \exp_{\lambda }\left(\theta \cdot F\left(\zeta \right)-\phi_{\lambda }\left(\theta \right)\right)\;d\mu .$$

This leads to:

$$\frac{\partial \phi_{\lambda }}{\partial \theta^{i}}=\int \tilde{p}\left(\zeta |\theta \right)F_{i}\left(\zeta \right)\;d\mu$$

with the escort transformation given by:

(36) $$\tilde{p}\left(\zeta |\theta \right)=\frac{{\left(p\left(\zeta |\theta \right)\right)}^{1-\lambda }}{\int {\left(p\left(\zeta |\theta \right)\right)}^{1-\lambda }\;d\mu }.$$

Clearly, when λ→0, we recover the regular exponential family (9). It was Tsallis who introduced the *q-exponential family*, where q=1−λ.

#### 5.2.2. Under Divisive Normalization

To deform the exponential family through divisive normalization, we use a smooth monotone function $\kappa_{\lambda }\left(\cdot \right)$ and define a parametric probability family, which takes the form:

$$\log p\left(\zeta |\vartheta \right)=\kappa_{\lambda }\left(\vartheta \cdot F\left(\zeta \right)\right)-\varphi_{\lambda }\left(\vartheta \right).$$

Note that we use the symbol ϑ to distinguish it from the parameter θ in the subtractive case. Here:

$$\varphi_{\lambda }\left(\vartheta \right)=\log\int e^{\kappa_{\lambda }\left(\vartheta \cdot F\left(\zeta \right)\right)}\;d\mu$$

is the divisive normalization function, and it was assumed that:

$$\int e^{\kappa_{\lambda }\left(\vartheta \cdot F\left(\zeta \right)\right)}d\mu <\infty$$

in the domain of ϑ (the natural parameter set). It is possible that the support of the density depends on the parameter ϑ, as in the case of the *q*-exponential family; see [17]. To avoid technicalities, we assumed that the support of p(ζ|ϑ) is independent of ϑ.

Writing out $\kappa_{\lambda }\left(\right)$, the resulting family is:

(37) $$p\left(\zeta |\vartheta \right)={\left(1+\lambda \vartheta \cdot F\left(\zeta \right)\right)}^{1/\lambda }\;e^{-\varphi_{\lambda }\left(\vartheta \right)},$$

where the divisive λ-potential $\varphi_{\lambda }\left(\vartheta \right)$ is given by:

(38) $$\varphi_{\lambda }\left(\vartheta \right)=\log\int {\left(1+\lambda \;\vartheta \cdot F\left(\zeta \right)\right)}^{1/\lambda }d\mu$$

is finite on the parameter set. This family unifies the *$F^{\left(\pm \alpha \right)}$-families* introduced in [12].

### 5.3. λ-Mixture Family

We next define a mixture-type family dual to the λ-exponential family, in an analogous way that an exponential family is dual to the mixture family. The form of the family is justified by its compatibility with the λ-duality.

**Definition** **5** (λ-mixture family)**.** *Let λ≠0,1 be given. The λ-mixture family with respect to a fixed set of densities $P_{0}\left(\zeta \right),P_{1}\left(\zeta \right),\ldots ,P_{d}\left(\zeta \right)$ is defined by:*

(39) $$p^{\left(\lambda \right)}\left(\zeta |\eta \right)=\frac{1}{Z_{\lambda }\left(\eta \right)}{\left(\sum_{i=0}^{d}\eta_{i}{\tilde{P}}_{i}\left(\zeta \right)\right)}^{1/(1-\lambda )},$$

*where $\eta =(\eta_{1},\cdots ,\eta_{d})$ is the mixture parameter satisfying $0\leq \eta_{i}\leq 1$ and $\eta_{0}=1-\sum_{i=1}^{d}\eta_{i}>0$. Here, ${\tilde{P}}_{i},\;i=0,1,\cdots ,d$ denotes the escort transformation, as given by *(36)*, of the given $P_{i}$’s:*

$${\tilde{P}}_{i}\left(\zeta \right)=\frac{{\left(P_{i}\left(\zeta \right)\right)}^{1-\lambda }}{\int {\left(P_{i}\left(\zeta \right)\right)}^{1-\lambda }\;d\mu },$$

*where the denominator is assumed to exist, and $Z_{\lambda }\left(\eta \right)$ represents the integral:*

$$\begin{array}{cc} Z_{\lambda }\left(\eta \right) & =\int {\left(\sum_{i=0}^{d}\eta_{i}{\tilde{P}}_{i}\left(\zeta \right)\right)}^{1/(1-\lambda )}\;d\mu , \end{array}$$

*which is assumed to converge for all η and to be differentiable under the integral sign.*

Denote:

$$C_{i}=\int {\left(P_{i}\left(\zeta \right)\right)}^{1-\lambda }d\mu$$

and:

$${\tilde{\eta }}_{i}=\frac{1}{h_{\lambda }\left(\eta \right)}\frac{\eta_{i}}{C_{i}}$$

where:

$$h_{\lambda }\left(\eta \right)=\sum_{i=0}^{d}\frac{\eta_{i}}{C_{i}}.$$

Then, $0\leq {\tilde{\eta }}_{i}\leq 1$ and $\sum_{i=0}^{d}{\tilde{\eta }}_{i}=1.$ We can express $p^{\left(\lambda \right)}$ now in $\tilde{\eta }$:

$$\begin{array}{cc} \; & p^{\left(\lambda \right)}=\frac{1}{Z_{\lambda }\left(\eta \right)}{\left(\sum_{i=0}^{d}\eta_{i}{\tilde{P}}_{i}\left(\zeta \right)\right)}^{1/(1-\lambda )}=\frac{h_{\lambda }\left(\eta \right)}{Z_{\lambda }\left(\eta \right)}{\left(\sum_{i=0}^{d}{\tilde{\eta }}_{i}{\left(P_{i}\left(\zeta \right)\right)}^{1-\lambda }\right)}^{1/(1-\lambda )}\\ \; & =e^{\log h_{\lambda }\left(\eta \right)-\log Z_{\lambda }\left(\eta \right)}{\left(\left(1-\sum_{i=1}^{d}{\tilde{\eta }}_{i}\right){\left(P_{0}\left(\zeta \right)\right)}^{1-\lambda }+\sum_{i=1}^{d}{\tilde{\eta }}_{i}\;{\left(P_{i}\left(\zeta \right)\right)}^{1-\lambda }\right)}^{1/(1-\lambda )}\\ \; & =e^{\log h_{\lambda }\left(\eta \right)-\log Z_{\lambda }\left(\eta \right)}{\left(1+\sum_{i=1}^{d}{\tilde{\eta }}_{i}\frac{{\left(P_{i}\left(\zeta \right)\right)}^{1-\lambda }-{\left(P_{0}\left(\zeta \right)\right)}^{1-\lambda }}{{\left(P_{0}\left(\zeta \right)\right)}^{1-\lambda }}\right)}^{1/(1-\lambda )}P_{0}\left(\zeta \right). \end{array}$$

Setting:

$$F_{i}\left(\zeta \right)=\frac{1}{1-\lambda }\left({\left(\frac{P_{i}\left(\zeta \right)}{P_{0}\left(\zeta \right)}\right)}^{1-\lambda }-1\right)\;,$$

with $d\nu =P_{0}\left(\zeta \right)d\mu$, the density of the λ-mixture family $p^{\left(\lambda \right)}$ with respect to the new measure ν now has the form:

$$p^{\left(\lambda \right)}\left(\zeta |\tilde{\eta }\right)={\left(1+\left(1-\lambda \right)\;\tilde{\eta }\cdot F\left(\zeta \right)\right)}^{1/(1-\lambda )}e^{-\psi_{1-\lambda }\left(\tilde{\eta }\right)},$$

where $\psi_{1-\lambda }\left(\tilde{\eta }\right)=\log Z_{\lambda }\left(\eta \right)-\log h_{\lambda }\left(\eta \right)$. Thus, we showed the following:

**Proposition** **6** (Relation between λ-exponential and λ-mixture families)**.** *Suppose λ≠0,1. A λ-mixture family with pure densities:*

$$P\left(\zeta \right)=\{P_{0}\left(\zeta \right),P_{1}\left(\zeta \right),\cdots ,P_{d}\left(\zeta \right)\}$$

*becomes a λ-exponential family with the vector of random functions:*

$$F\left(\zeta \right)=\{F_{1}\left(\zeta \right),\cdots ,F_{d}\left(\zeta \right)\}$$

*after a transformation of the dominating measure $d\mu \to d\nu =P_{0}\left(\zeta \right)d\mu$ and the random variables P(ζ)→F(ζ):*

$$F_{i}\left(\zeta \right)=\frac{1}{1-\lambda }\left({\left(\frac{P_{i}\left(\zeta \right)}{P_{0}\left(\zeta \right)}\right)}^{1-\lambda }-1\right)=\log_{1-\lambda }\left(\frac{P_{i}\left(\zeta \right)}{P_{0}\left(\zeta \right)}\right)\;,$$

*and a reparameterization $\eta \to \tilde{\eta }$:*

$${\tilde{\eta }}_{i}=\frac{1}{h_{\lambda }C_{i}}\eta_{i}⟺\eta_{i}={\tilde{\eta }}_{i}\left(h_{\lambda }C_{i}\right)$$

*with:*

$$h_{\lambda }=\sum_{i=0}^{d}\frac{\eta_{i}}{C_{i}}=\sum_{i=0}^{d}{\tilde{\eta }}_{i}C_{i}.$$

### 5.4. Potential Functions as Rényi Entropies

We now show that our λ-duality framework is naturally compatible with the λ-exponential and λ-mixture families, with Rényi entropy and Rényi divergence replacing Shannon entropy and Kullback–Leibler divergence. In what follows, we assume λ<1.

**Proposition** **7** (For the λ-exponential family)**.** *With respect to the λ-exponential family defined by *(37)* with divisive potential function $\varphi_{\lambda }$ given by *(38)*, we have:*

*(i)*  *$\varphi_{\lambda }\left(\vartheta \right)$ is λ-convex. Moreover, $1-\lambda D\varphi_{\lambda }\left(\vartheta \right)\cdot \theta >0$.*
*(ii)*  *The λ-conjugate variable $\eta =D^{\left(\lambda \right)}\varphi_{\lambda }\left(\vartheta \right)=D\phi_{\lambda }\left(\theta \right)$ is the the escort expectation:*
  $$\eta =\frac{\int {\left(p\left(\zeta |\theta \right)\right)}^{1-\lambda }\;F\left(\zeta \right)\;d\mu }{\int {\left(p\left(\zeta |\theta \right)\right)}^{1-\lambda }\;d\mu }=\int \tilde{p}\left(\zeta |\theta \right)\;F\left(\zeta \right)\;d\mu .$$
*(iii)*  *The λ-conjugate function $\psi_{\lambda }\left(\eta \right)$ with respect to $\varphi_{\lambda }\left(\vartheta \right)$ is given by:*
  $$\psi_{\lambda }\left(\eta \right)=-H_{\lambda }^{\mathrm{Rényi}}\left[p\left(\cdot |\vartheta \right)\right].$$
*(iv)*  *The λ-logarithmic divergence is the Rényi divergence:*
  $$L_{\lambda ,\varphi_{\lambda }}\left(\vartheta ,\vartheta^{\prime }\right)=H_{\lambda }^{\mathrm{Rényi}}[p\left(\cdot |\vartheta^{\prime }\right)||p\left(\cdot |\vartheta \right)].$$

**Proposition** **8** (For the λ-mixture family)**.** *With respect to the λ-mixture family given by *(39)* with its potential function $\psi_{\lambda }\left(\eta \right)$ given by:*

$$\psi_{\lambda }\left(\eta \right)=\frac{1-\lambda }{\lambda }\log\int {\left(\sum_{i=0}^{d}\eta_{i}{\tilde{P}}_{i}\right)}^{1/(1-\lambda )}d\mu =\frac{1-\lambda }{\lambda }\log Z_{\lambda }\left(\eta \right),$$

*we have:*

*(i)*  *The potential function $\psi_{\lambda }\left(\eta \right)$ is a λ-convex function of η.*
*(ii)*  *The potential function $\psi_{\lambda }\left(\eta \right)$ is given by:*
  $$\psi_{\lambda }\left(\eta \right)=-H_{\lambda }^{\mathrm{Rényi}}\left[p\left(\cdot |\eta \right)\right].$$
*(iii)*  *The λ-logarithmic divergence is the Rényi divergence:*
  $$L_{\lambda ,\psi_{\lambda }}\left(\eta ,\eta^{\prime }\right)=H_{\lambda }^{\mathrm{Rényi}}[p\left(\cdot |\eta \right)||p\left(\cdot |\eta^{\prime }\right)].$$

The proofs of the above Proposition 7 (about the λ-exponential family) and Proposition 8 (about the λ-mixture family) can be found in [17].

## 6. Summary and Conclusions

Our paper summarizes a canonical approach to deforming exponential and mixture families and the associated dually flat Hessian geometry. The λ-exponential family we introduced has two parameterizations (35) and (37):

$$p^{\left(\lambda \right)}\left(\zeta |\cdot \right)=\exp_{\lambda }\left(\vartheta \cdot F\left(\zeta \right)\right)e^{-\varphi_{\lambda }\left(\vartheta \right)}=\exp_{\lambda }\left(\theta \cdot F\left(\zeta \right)-\phi_{\lambda }\left(\theta \right)\right).$$

The two expressions reflect subtractive and divisive normalizations—a typical example of the former is the *q*-exponential family with associated Tsallis entropy, whereas an example of the latter is the $F^{\left(\pm \alpha \right)}$-family and the associated Rényi entropy. These two versions of deformation to an exponential family are two faces of the same coin; furthermore, the λ-exponential family is also linked to the λ-mixture family, when λ≠0,1, via a reparameterization of the random functions F(ζ) above.

The coincidence of these two parameterizations of the deformed family is associated with the λ-duality, which is the main focus of our exposition. The λ-duality is a “deformation” (see Table 1) of the usual Legendre duality reviewed in Section 3.1. In a nutshell, instead of convex functions, we worked with λ-convex functions *f* such that $\frac{1}{\lambda }\left(e^{\lambda f}-1\right)$ is convex, for a fixed λ≠0. Furthermore, instead of the convex conjugate, we used the λ-conjugate given by:

$$f^{\left(\lambda \right)}\left(u\right)=\underset{x}{\sup}\left(\frac{1}{\lambda }\log\left(1+\lambda x\cdot u\right)-f\left(x\right)\right).$$

The expression of the λ-duality:

$$\kappa_{\lambda }\left(x\cdot u\right)=f\left(x\right)+f^{\left(\lambda \right)}\left(u\right),$$

turns out to be a re-write of the Legendre duality between $\tilde{x}$ and *u*:

$$\tilde{x}\cdot u=g\left(\tilde{x}\right)+g^{*}\left(u\right)\;,\;\;\;\;\;\;\;\;\;with\;\;\;\;\tilde{x}=xe^{-\lambda f(x)};$$

and a re-write of the Legendre duality between *x* and $\tilde{u}$:

$$x\cdot \tilde{u}={\tilde{g}}^{*}\left(x\right)+\tilde{g}\left(\tilde{u}\right)\;,\;\;\;\;\;\;\;\;\;\;with\;\;\;\;\tilde{u}=ue^{-\lambda f^{\left(\lambda \right)}\left(u\right)}.$$

Therefore, λ-duality is in essence the Legendre duality with a λ-dependent rescaling of the variables:

$$\tilde{x}=x\;e^{-\lambda \;f(x)}⟺x=\frac{\tilde{x}}{1-\lambda g(\tilde{x})}$$

and:

$$\tilde{u}=u\;e^{-\lambda \;f^{\left(\lambda \right)}\left(u\right)}⟺u=\frac{\tilde{u}}{1-\lambda \tilde{g}\left(\tilde{u}\right)}.$$

The two pairs of convex functions $g,g^{*}$ and $\tilde{g},{\tilde{g}}^{*}$ are linked with the pair of λ-convex functions $f,f^{\left(\lambda \right)}$ via:

$$\begin{array}{ccc} g(\tilde{x}) & = & G_{-\lambda ,f}^{}\left(x\right)=\gamma_{-\lambda }\left(f\left(x\right)\right)=\left(\gamma_{-\lambda }\circ f\right)\left(x\right);\\ g^{*}\left(u\right) & = & G_{\lambda ,f^{\left(\lambda \right)}}^{}\left(u\right)=\gamma_{\lambda }\left(f^{\left(\lambda \right)}\left(u\right)\right)=\left(\gamma_{\lambda }\circ f^{\left(\lambda \right)}\right)\left(u\right);\\ \tilde{g}\left(\tilde{u}\right) & = & G_{-\lambda ,f^{\left(\lambda \right)}}^{}\left(u\right)=\gamma_{-\lambda }\left(f^{\left(\lambda \right)}\left(u\right)\right)=\left(\gamma_{-\lambda }\circ f^{\left(\lambda \right)}\right)\left(u\right);\\ {\tilde{g}}^{*}\left(x\right) & = & G_{\lambda ,f}^{}\left(x\right)=\gamma_{\lambda }\left(f\left(x\right)\right)=\left(\gamma_{\lambda }\circ f\right)\left(x\right). \end{array}$$

The λ-duality leads to nontrivial mathematical questions, e.g., a differential calculus in the spirit of Rockafellar and analogous to functions of the Legendre type. Some of the derivations in the current paper were heuristic, and a complete and rigorous development is left for future research.

Coming back to the probability families, we first verified that the subtractive potential $\phi_{\lambda }\left(\theta \right)$ is convex in θ and the divisive potential $\varphi_{\lambda }\left(\vartheta \right)$ is λ-convex in ϑ. Subtractive normalization using $\phi_{\lambda }\left(\theta \right)$ is associated with the regular Legendre duality, whereas divisive normalization using $\varphi_{\lambda }\left(\vartheta \right)$ is associated with the λ-duality. This gives an interpretation of the distinctiveness of Rényi entropy (used in the latter) from Tsallis entropy (used in the former) based on their intimate connection to the λ-duality (for λ≠0) or to the Legendre duality. As λ is the parameter that controls the curvature in the Riemannian geometry of these probability families (see [12]), our framework provides a simple parametric deformation from the dually flat geometry (of the exponential model) to the dually projectively flat geometry (of the λ-exponential model). We expect that this framework will generate new insights in the applications of the *q*-exponential family and related concepts in statistical physics and information science.

## Figures and Tables

**Table 1:** Generalization of objects from the Hessian (dually flat) geometry to the λ-deformed (dually projectively flat) geometry.

Objects	Conventional (λ=0)	λ-Deformed
transformation	Legendre	λ-Legendre
conjugation	$\sup_{x}\left(x\cdot u-f\left(x\right)\right)$	$\sup_{x}\left(\kappa_{\lambda }\left(x\cdot u\right)-f\left(x\right)\right)$
potentials	convex	λ-convex
associated divergence	Bregman	λ-logarithmic
Riemannian metric	Hessian	conformal Hessian
affine connections	dually flat	dually projectively flat
curvature of connections	0	constant λ≠0
biorthogonal coordinates	(x,u)	$\left(\tilde{x},u\right)$ or $\left(x,\tilde{u}\right)$
probability family	exponential	λ-exponential
probability family	mixture	λ-mixture
associated divergence	Kullback–Leibler	Rényi
associated entropy	Shannon	Rényi/Tsallis

## Data Availability

Not applicable.
